# Evaluating women’s experiences and satisfaction with labour induction in India: a comparison of the participant generated experience and satisfaction (PaGES) index with standard methods

**DOI:** 10.1186/s12884-025-07731-9

**Published:** 2025-05-28

**Authors:** Avni Patel, Rachel Howard, Brian Faragher, Jill Durocher, Beverly Winikoff, Andrew Symon, Andrew Weeks, Shuchita Mundle, Kate Lightly

**Affiliations:** 1 University of Liverpool, Liverpool, L69 3BX UK; 2 Liverpool School of Tropical Medicine, Pembroke Place, Liverpool, L3 5QA UK; 3 Gynuity Health Projects, 215 Lexington Ave, Suite 1702, New York, NY 10017 USA; 4 Mother and Infant Research Unit, University of Dundee, 11 Airlie Place, Dundee, DD1 4HJ Dundee, UK; 5 Department of Women’s and Children’s Health, University of Liverpool, Liverpool Women’s Hospital, Crown Street, Liverpool, L8 7SS UK; 6 Department of Obstetrics and Gynaecology, All India Institute of Medical Sciences, Nagpur, India; 7 University of Liverpool, Liverpool Women’s Hospital, Crown Street, Liverpool, L8 7SS UK

**Keywords:** Participant, Experience, Satisfaction, Patient-generated, Qualitative research, Quantitative research, Induction of labour, Instrument, Birth, Patient reported experience measure, Patient reported outcome measure.

## Abstract

**Background:**

Although induction of labour is becoming more common worldwide, there are few studies that assess women’s satisfaction with it. The newly developed Participant Generated Experience and Satisfaction (PaGES) Index collects brief qualitative data and quantifies it, allowing detailed satisfaction data to be collected on large populations. The PaGES data has never previously been compared to other methods of assessing study participants’ satisfaction. We aimed to triangulate PaGES Index, Likert questionnaire and interview data from a large, randomised trial of labour induction to fully understand women’s priorities, experience and satisfaction and to compare the findings of the three instruments.

**Methods:**

A convergent parallel multi-methods research design was used. Participants in the Misoprostol or Oxytocin for Labour Induction (MOLI) trial (*n* = 520) completed the PaGES Index before and after birth, listing priorities and allocating spending points to demonstrate their relative importance. Postpartum, participants scored their satisfaction with each item. Quantitative data was collected following birth on the acceptability of augmentation, delivery time, pain and anxiety using a Likert scale. Semi-structured interviews were also conducted, and thematic analysis was carried out using a framework approach. The data from 20 participants who had completed all three outcome measures were integrated and compared.

**Results:**

Although common themes, such as pain, emerged from participants’ responses to the three instruments, each provided different insights. The Likert responses demonstrated overall satisfaction with the induction process but with high levels of pain and anxiety. Semi-structured interviews highlighted that safety and health of the baby was a key priority. The PaGES Index confirmed that the baby’s wellbeing was most important to women, but women also expressed a strong preference for vaginal delivery.

**Conclusions:**

The PaGES Index, Likert questionnaire and semi-structured interview data provide varied but complimentary insights on women’s birth experiences and their satisfaction with their induction process. The outputs of the three methods align, but the PaGES index was unique in capturing both detailed qualitative and quantitative information for all study participants.

**Trial registration:**

The MOLI study is registered in ClinicalTrials.gov (NCT03749902, Registration date: 21st Nov 2018) and Clinical Trial Registry, India (CTRI/2019/04/018827).

**Supplementary Information:**

The online version contains supplementary material available at 10.1186/s12884-025-07731-9.

## Background

Evaluating patient experience and satisfaction in clinical trials is vitally important and can be achieved through quantitative, qualitative and combined qualitative and quantitative instruments [1]. This paper evaluates all three approaches within a multicentre randomised controlled trial (RCT) of labour induction methods in India.

Induction of labour (IOL), defined as “the artificial stimulation of the uterus to initiate labour” [2], is a common practice in India [3, 4]. Research conducted in high-income settings has yielded mixed findings regarding the impact of IOL on women’s childbirth experiences. While some studies suggest that IOL is not associated with negative childbirth outcomes [5, 6], others report that women who undergo induction tend to have more negative experiences compared to those with spontaneous labour onset [7]. Factors such as inadequate information and insufficient support during the induction process can contribute to increased anxiety and a diminished sense of control, ultimately leading to a more negative experience [8]. Women may feel concerned about the potential risks to their own or their baby’s health, and report feelings of helplessness and disappointment [9]. However, despite the importance of maternal satisfaction, fewer than 5% of studies in a network meta-analysis of IOL methods provided data on this aspect [10].

A systematic review identified 36 existing tools for evaluating birth experience and satisfaction [11]. Of these, only seven were found to be sufficiently reliable [12–18]. The Wijma Delivery Expectancy/Experience Questionnaire rated highest for both reliability and validity [18]. However, no tool was able to provide both qualitative and quantitative data. Another review identified nine questionnaires [19] with two standard questionnaires, Perceptions of Care Adjective Checklist [14] and Six Simple Questions [13], recommended as potentially useful tools for comparing satisfaction at various time points (see appendix 1).

The MOLI RCT recruited 1,033 women undergoing induction for hypertensive disorders of pregnancy. The study compared the effectiveness of using a low dose oral misoprostol regimen for cervical ripening and augmentation after artificial rupture of membranes with the standard protocol of intravenous oxytocin following cervical ripening with oral misoprostol [20]. The full MOLI trial results were recently published by Mundle et al. [21].

The study’s aims also encompassed the assessment of the protocols’ safety, efficacy and acceptability, showing comparable results between the two methods. Data relating to women’s satisfaction was collected through Likert scale satisfaction questionnaires and an alongside qualitative sub-study involving semi-structured interviews with a sample of participants [22].

A novel patient-reported outcome measure (PROM), the Participant-Generated Experience and Satisfaction (PaGES) Index was also incorporated in the study [23]. The PaGES index was developed from the Patient Generated Index (PGI), a self-administered three-part questionnaire which had patients (a) identify important areas of quality of life affected, (b) rate how badly they are affected by each, and then (c) allocate spending points to demonstrate the need for improvement in each domain [24]. This tool has now been widely utilised and validated across a range of medical disciplines [25–27]. The Mother-Generated Index (MGI), a modified form of the PGI was designed to evaluate quality of life in women following childbirth [28]. Nagpal et al. were the first to conduct a study exploring the use of the MGI in India [29]. The researchers identified a clear trend of lower quality of life scores amongst women of lower socioeconomic status. However, only 195 of 282 eligible patients in the study could be assessed due to issues such as poor comprehension and refusal to consent. The PaGES Index is a new combined qualitative and quantitative quality of life evaluation tool, developed from the MGI, that was piloted in the MOLI study. In previous work, the concept of ranking priorities had posed challenges, particularly for less educated groups, due to difficulties in understanding the mathematical aspects involved. To help women better understand this concept, 20 cooking beans were used, a stratagem adapted from a study in Brazil where participants struggled to conceptualise ‘spending points’ but were familiar with distributing a food item [30].

The MOLI study protocol and a methods paper introducing the PaGES Index have been published previously [20, 23]. This paper reports women’s experiences of induction of labour in the MOLI study and compares the PaGES index to standard methods of data collection to determine the optimum method for understanding women’s priorities, experiences, and satisfaction in IOL trials.

## Methods

1033 women requiring IOL were recruited into the trial across three public hospitals in Maharashtra, India: Government Medical College in Nagpur (a tertiary centre with 8,615 births recorded in 2023), Mahatma Gandhi Institute of Medical Sciences in Sevagram (a rural teaching hospital with ~ 3500 births recorded in 2023), and Daga Memorial Hospital in Nagpur (a Women’s hospital with 10,135 births recorded in 2023) between January 2020 and July 2022. Informed consent was sought once the decision for induction was made. The complete inclusion and exclusion criteria for participants on the MOLI trial are available in the registered MOLI protocol on ClinicalTrials.gov (NCT040037683), and the clinical outcomes are published in a separate report [31].

**Fig. 1:**
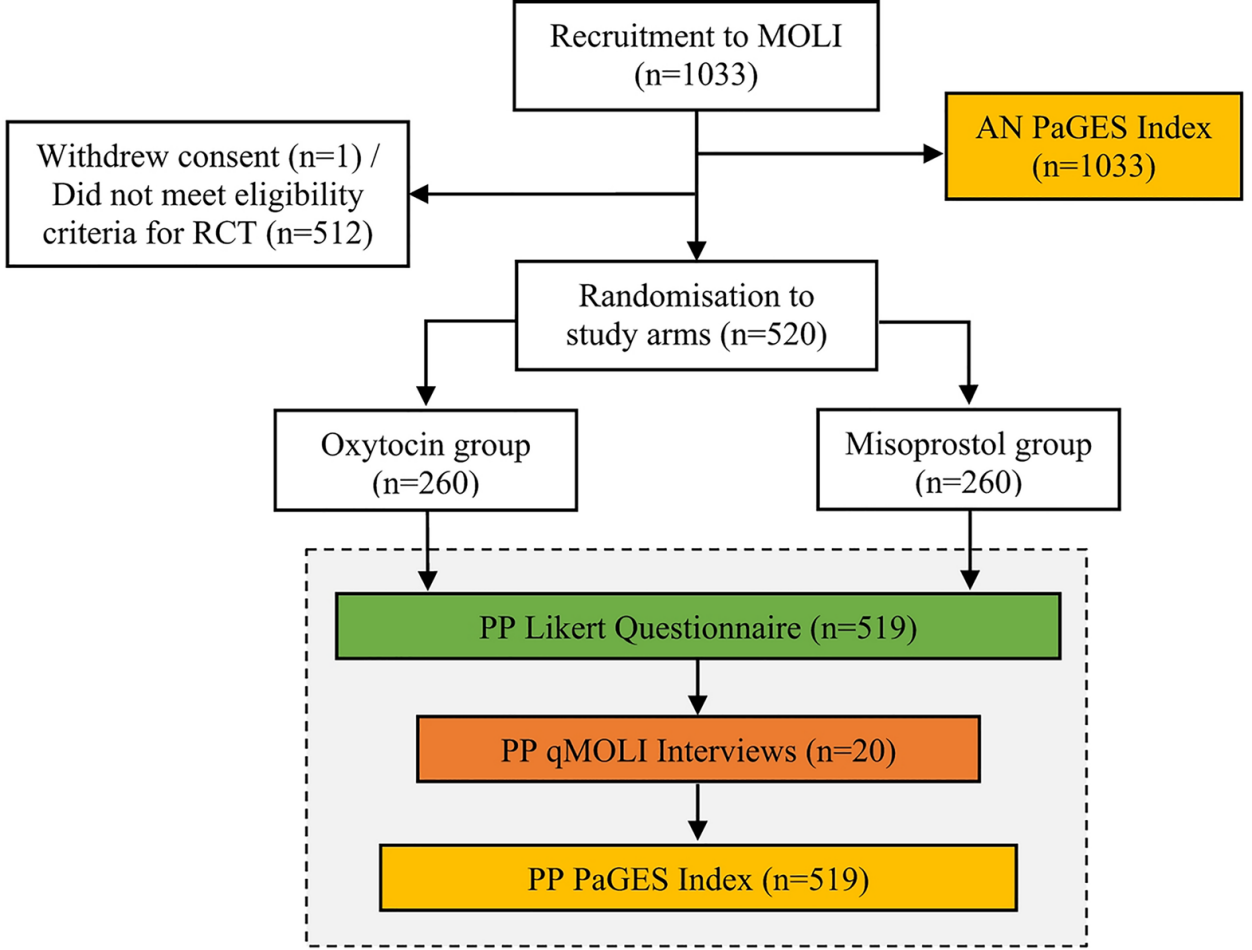
Misoprostol or Oxytocin for Labour Induction (MOLI) randomised control trial study flow adapted from MOLI study protocol [20]. The grey box highlights the data used to understand women’s experience and satisfaction following induction and birth. The PaGES Index was completed by 519 of the 520 women in the MOLI RCT. 519 women in the RCT also completed the Likert scale questionnaire following birth. A sample of women (*n* = 20) were also interviewed 1–6 days postpartum. *Abbreviations*: AN– antenatal, PP– postpartum, MOLI– Misoprostol or Oxytocin for Labour Induction, qMOLI– qualitative MOLI sub-study, PaGES Index– Patient Generated Experience and Satisfaction Index

All women recruited into the RCT were initially given low-dose oral misoprostol for cervical ripening, following this, 520 women requiring additional augmentation were randomised to receive either continued oral misoprostol or intravenous oxytocin (Fig. 1). Participants and their babies were followed up until hospital discharge.

The Likert scale questionnaires, postpartum participant interviews and postpartum PaGES Index data were generally collected in Hindi or Marathi by a trained research associate (RA). The Likert scale responses were entered into the online data collection tool. The interviews were recorded, transcribed and translated, whilst the PaGES responses were recorded on paper in Hindi or Marathi for later translation into English. The results were integrated through a convergent parallel multi-methods design, whereby data was collected and analysed independently, and the results interpreted together. The data from participants who completed all three tools were synthesised in a summary table. Key findings and overlapping areas between the three outcome measures were also displayed diagrammatically.

### Likert scale questions

Four Likert questions were incorporated in the MOLI post-delivery follow-up questionnaire administered by research associates within 24 h of the birth. Women were asked to rate the acceptability of augmentation and delivery time, as well as the amount of pain and anxiety on 5-point scales. To allow for comparison to the PaGES postpartum satisfaction scores, the pain and anxiety scales were inverted and given values from 0 to 10, with 10 representing highest satisfaction and 0 lowest satisfaction (Table 1).

**Table 1:** Likert scale questions asked during exit interviews and response codes

Domain assessed	Question	Likert scale (value given for comparative scoring purposes)
Augmentation	Please ask the woman to rate the acceptability of the augmentation method	Very acceptable (10) Acceptable (7.5) Neutral (5) Unacceptable (2.5) Very unacceptable (0)
Delivery time	Please ask the woman to rate the acceptability of the time taken for her delivery	Very acceptable (10) Acceptable (7.5) Neutral (5) Unacceptable (2.5) Very unacceptable (0)
Pain	Please ask the woman to rate the amount of pain experienced during her induction and delivery	None (10) Slight (7.5) Moderate (5) High (2.5) Extreme (0)
Anxiety	Please ask the woman to rate the amount of anxiety experienced during her induction and delivery	None (10) Slight (7.5) Moderate (5) High (2.5) Extreme (0)

### qMOLI study

53 semi-structured interviews were conducted between January 2021 and July 2022 with women enrolled in the MOLI study either pre-IOL (*n* = 19) or 1–6 days postpartum (*n* = 34). Prior to recruitment, a sampling framework was developed to ensure data collection from a diverse group of women with varied experiences. The framework outlined key characteristics, such as parity, mode of birth and induction method, to create a cohort of women whose demographics reflected those of the randomised MOLI participants. Interviews continued until data saturation was met.

During the postpartum interviews, women were asked questions such as;

- Can you explain what things are important to you about this process?
- Of these things (mentioned in last question), what is the most important thing?
- Is there anything that you feel particularly positive/happy about this process?

Interviews were conducted in Hindi or Marathi by a trained research associate (RA) and were recorded, transcribed and translated. Transcripts and translation were counter-checked by a second RA.

Thematic analysis of interview data from the subset of participants who were postpartum and had been randomised in the MOLI study (*n* = 20) was carried out in NVivo 20 using a framework approach [32]. Initially, the researchers familiarised themselves with the transcripts through repeated readings. Data were then openly coded to a working analytical framework by two researchers (KL and LH) who worked independently on the data. Any discrepancies or differences in interpretation between the two analysts were resolved through discussion with the wider research team. Following the coding of the transcripts, the researchers conducted several iterations of analysis to refine and consolidate the emerging themes. The final codes were aggregated into overarching themes that represented the core aspects of participants’ experiences and perspectives.

### PaGES index

**Fig. 2:**
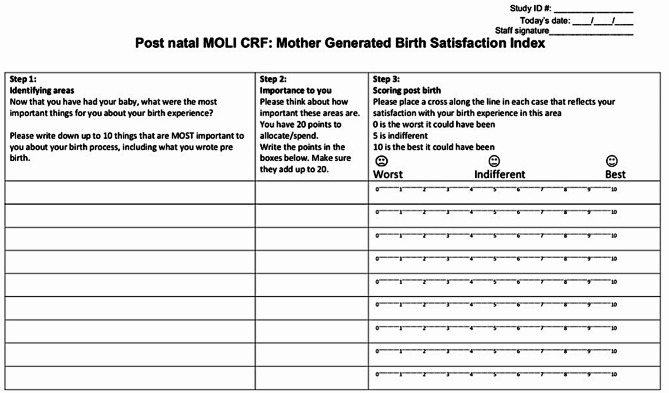
Postpartum PaGES Index case report form (CRF). Women identify up to 10 important areas post-IOL and allocate 20 spending points to demonstrate the personal importance of these areas. Step 3 allows women to rate their satisfaction with their birth experience in each of the areas they have identified as important to them

519 women recruited into the MOLI randomised trial also completed the PaGES Index after birth alongside the Likert questionnaire. Women first listed their priorities, either by writing them down themselves or with the assistance of the research assistant (RA). They were then given 20 dried kidney beans and instructed to allocate them as “spending points” across their priorities to reflect their relative importance. The RA or the woman herself recorded the number of beans allocated to each priority, according to her preferences (Fig. 2). The PaGES tool was also administered antenatally at recruitment (see appendix 2).

A coding framework was developed in accordance with the framework approach following an internal pilot study [32]. PaGES statements were inductively and independently coded using line-by-line thematic coding by one researcher (RH) and checked by a second researcher (AP). The data were coded with supervision from KL and with oversight from the trial management group. As with the interview data, coding was repeated using multiple iterations of the coding framework and any discrepancies were resolved through discussion with the wider team. The finalised codes were aggregated into themes and overarching themes.

Statistical analysis was carried out in SPSS 28.0.1.1. In this paper those recruited to the MOLI study are treated as a single cohort (the comparative PaGES results from the two arms will be published elsewhere). The number of women citing each theme was evaluated. As many mothers cited more than one important issue within the same theme, the total number of women citing each theme and overarching theme was calculated. The relative importance (number of allocated spending points) of each theme and satisfaction scores were reported as means and standard deviations. An overall PaGES score for each woman was generated by totalling the multiplied postpartum bean and satisfaction scores allocated to each of the participant’s statements.

### Comparative analyses

Comparative analyses were conducted by integrating postpartum data from 20 participants who completed all three tools (Likert scale, PaGES Index, and qMOLI interview). Patterns and inconsistencies were identified through descriptive statistics and qualitative content analysis to assess alignment between quantitative and qualitative measures.

## Results

### Likert scale questions

**Fig. 3:**
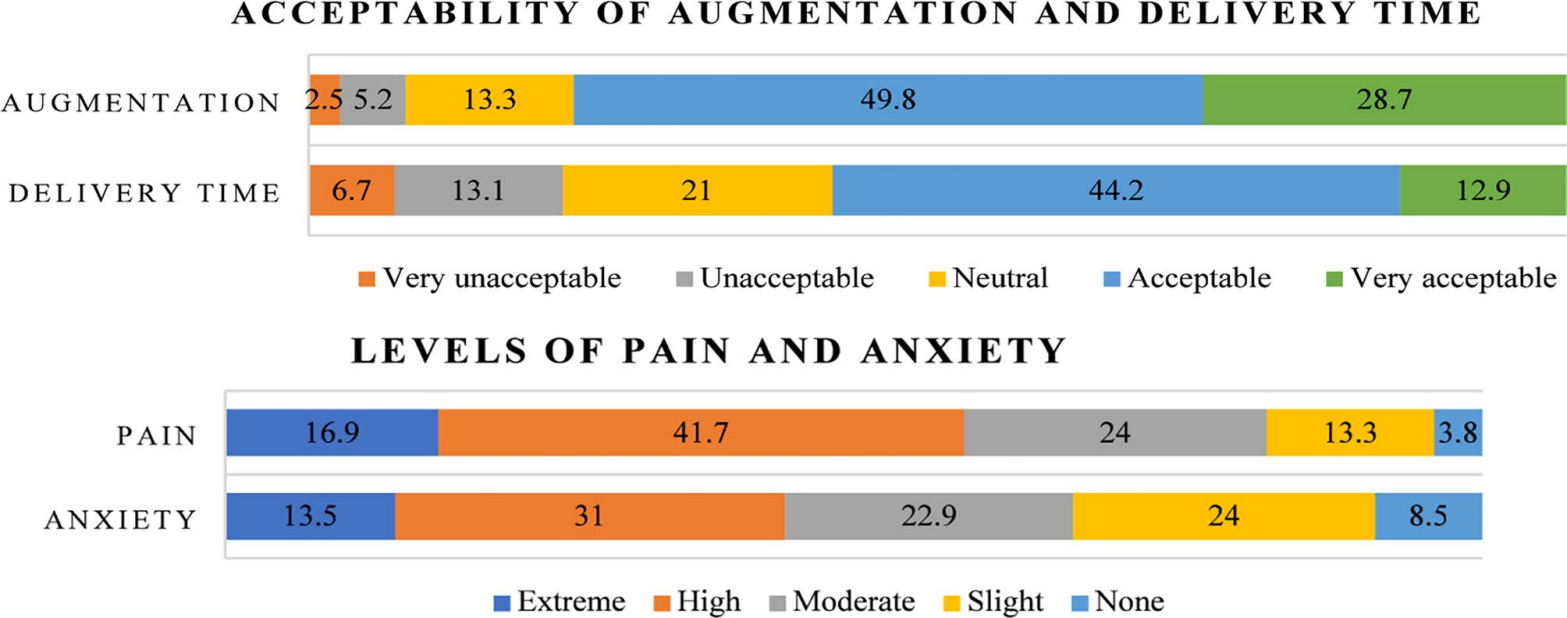
Distribution of Likert question responses. Stacked bar charts illustrating the percentage of responses to the four Likert scale questions regarding the acceptability of augmentation, delivery time, pain and anxiety

519 of the 520 women randomised in the MOLI trial completed the Likert questionnaire. One woman failed to complete the exit questionnaire and was excluded from analysis. Women reported overall satisfaction with the induction process and delivery. 78.5% of women said the overall augmentation process was acceptable (49.8%) or very acceptable (28.7%) and 57.1% of women said their delivery time was acceptable (44.2%) or very acceptable (12.9%). Over half of women reported high or extreme pain (58.6%) and 44.5% reported high or extreme anxiety (Fig. 3).

### qMOLI study

53 interviews were conducted pre- or post-IOL with women from a wide range of socioeconomic classes and varied educational status. Twenty interviews were carried out postpartum with women who had been randomised and administered either misoprostol (*n* = 9) or oxytocin (*n* = 11). For the study aims of this paper, this subgroup of interviews was analysed to understand women’s experiences, priorities and satisfaction in the MOLI trial.

This cohort of qMOLI data is described in two themes. *Women’s experiences of IOL and childbirth* and *Important areas identified by women*. *Women’s experiences of IOL and childbirth* is split into the following subthemes: “blood pressure”, “delivery”, “environment”, “family”, “IOL process”, “knowledge”, “pain/*traas*”, “staff” and “thoughts and feelings”. *Important areas identified by women* encompasses: “baby’s future, “baby’s gender”, “timely birth and discharge”, “mode of birth”, “no pain/*traas*”, “own health” and “safety and health of baby”. *‘Traas’* is a Marathi term that encompasses both mental and physical distress or suffering, and it was a key sentiment that emerged from the qMOLI interviews. It was not translated directly to preserve the cultural and emotional nuance captured in the participants’ responses. Table 2 illustrates the coding framework and illustrative quotes from the analysis of the 20 postpartum qMOLI interviews with randomised participants. Additional quotes can be found in appendix 3.

**Table 2:** qMOLI coding framework. Themes and subthemes of qMOLI interview thematic analysis with illustrative quotes

Theme	Subtheme	Illustrative quotes
Women’s experiences of IOL and childbirth	Blood pressure	“Only was worrying about BP” (Interview 3)
	Childbirth	“As soon as I got there, I had to push hard. Doctor was asking me to push, I pushed hard and delivered normally.” (Interview 31)
	Environment	“I was seeing the ladies, so I was more scared. (Interview 11)
	Family	“My family members are happy. Specially my husband is very happy.” (Interview 46)
	IOL process	“They had given the first pill. It didn’t bring the pain. Given a second pill, there was a backache. After the third pill, I had more pain.” (Interview 37)
	Knowledge	“I don’t have knowledge about it… but I heard from my friend, “I went to the hospital, and I delivered normally within half an hour”. If she delivers normally then why not me?” (Interview 45)
	Pain / ‘traas’	“What a pain… the pain was so much… I was feeling it’s coming every 2–3 minutes… such a pain was there… a lot of pain, lot of pain.” (Interview 46)
	Staff	“And then […] madam was there. She helped me a lot.” (Interview 46)
	Thoughts and feelings	“Nervousness is there na… How will it happen, what will happen? (Interview 18)
Important areas identified by women	Baby’s future	“Yes… like I became a mother… how will be my baby? What will he do in future?” (Interview 37)
	Baby’s gender	“There was no tension, whether it’s a baby girl or male.” (Interview 3)
	Mode of birth	“Caesar don’t have trouble early but afterwards there is a lot of trouble… normal delivery is good.” (Interview 8)
	Timely birth and discharge	“Now, waiting to go home. When it will discharge (smiled).” (Interview 10)
	Own health	“My BP should not raise.” (Interview 10)
	No pain / ‘traas’	“I was feeling that there should not be much pain.” (Interview 10)
	Safety and health of baby	“My baby should be safe… I would have done anything for that.” (Interview 16)

#### Women’s experiences of IOL and childbirth

Women discussed nine focal areas of the IOL experience. They acknowledged that elevated **blood pressure** was the primary reason for induction and recognised its potential risk to their baby’s health. Anxiety, perceived as a key contributor to hypertension, raised concerns among women, with some fearing the possibility of a caesarean section if blood pressure remained high. **Environmental** factors, including witnessing other women’s struggles during **childbirth**, negatively influenced some participants. **Fear and anxiety** were prevalent emotions during the IOL process, particularly among primiparous women. **Family** advice and comparisons with relatives’ childbirth experiences were common topics of discussion. Limited prior **knowledge** about IOL methods was noted, and several women were under the impression that the use of “saline” (oxytocin) would result in faster delivery. Despite apprehensions, women generally appreciated the necessity of induction for maternal and foetal safety. Some struggled to distinguish between **IOL** and the overall childbirth experience. Pain expectations varied, and contractions were often described as “terrible,” after misoprostol was commenced. Positive relationships with **staff**, feeling supported and well-cared-for, and respectful interactions with healthcare providers were highlighted. Women **thought/felt** participation in the MOLI trial contributed to better quality care.

### Important areas identified by women

Women identified seven key priorities during discussions. They often detailed plans for the **baby’s future**, emphasising the importance of the baby remaining well, with considerations for feeding, care, and education. **Gender** preferences were mentioned by around half of the women, with few expressing disappointment, often deferring to male family members’ opinions. Despite a preference for vaginal delivery, women prioritised the safe birth of their **baby** over the **mode of birth**, with positive sentiments about vaginal birth and some expressing relief after the fact. **Timely birth and discharge** were crucial for some, while concerns about **pain and “traas”** during labour were common priorities. **Own health** issues such as maintenance of normal blood pressure and discussions about family planning operations were also highlighted. Overall, the **safety and health of the baby** emerged as the foremost priority for almost all women, often evoking strong emotional responses.

### PaGES index

2755 statements were made by the 519 women who completed the postpartum PaGES Index form, of which 1966 were allocated spending points (Table 3). All postpartum codes were organised into subthemes and seven overarching themes. Overarching themes identified were “perspective on the birth”, “perspective on the baby”, “mother’s emotional wellbeing”, “looking to the future”, “family”, “mother’s physical health” and “miscellaneous”. “Satisfaction with normal vaginal delivery” (*n* = 183) and “baby is healthy” (*n* = 209) were the most frequently stated postpartum codes.

**Table 3:** Postpartum pages codes. The top 10 most frequently cited postpartum pages codes with number and percentage of participants citing the code. The importance and satisfaction scores for each theme are summarised in Table 4. Statements within the “perspective on the birth” overarching theme were allocated the highest proportion of spending points followed by statements relating to “perspective on the baby”. “Mother’s physical health” statements were allocated the fewest

Code	Number of codes allocated beans (% of participants)
Baby– healthy	212 (40.8%)
Mode of Delivery– satisfaction with Normal Vaginal Delivery	179 (34.4%)
Family– happy/good	140 (26.9%)
Happy– to have baby	145 (27.9%)
Postpartum– discharge	73 (14.0%)
Postpartum– care for baby	124 (23.8%)
Everything was good– staff	87 (16.7%)
Pain– general	50 (9.6%)
Gender– important	88 (16.9%)
Long-term future– baby	77 (14.8%)
**Total codes**	**1966 (71.4%)**

**Table 4:** Summary of pages theme results. Number of women citing each theme and mean postpartum importance (spending point) scores* for all participants who cited each overarching theme with standard deviations (SD). The “miscellaneous” theme was intentionally omitted from the table as it contained a diverse range of poorly defined responses that did not align with the other more distinct themes. *If a woman cited an overarching theme more than once her scores have been averaged to generate a mean bean score with a numerator where n = number of women. The mean satisfaction scores for each overarching theme and study group are shown in Table 5. Mean postnatal satisfaction levels ranged from 5.19 for “mother’s physical health” to 9.03 for “family”. Statistically significant differences in satisfaction scores between the misoprostol and oxytocin study groups were observed for “looking to the future” (7.31 vs. 7.03, *p* = 0.049), “mothers physical health” (5.77 vs. 4.68, *p* = 0.032) and “perspective on baby” (8.88 vs. 8.51, *p* = 0.024), all of which had higher satisfaction in the misoprostol group

	Number (and %) of women citing the theme	Importance– mean number of spending points allocated (+/- SD)
Perspective on birth	431 (82.9%)	8.3 (+/- 5.31)
Perspective on baby	358 (68.9%)	7.17 (+/- 4.29)
Mother’s emotional wellbeing	210 (40.4%)	5.08 (+/- 3.85)
Looking to the future	396 (76.2%)	4.96 (+/- 4.49)
Family	180 (34.6%)	4.05 (+/- 3.47)
Mother’s physical health	84 (16.2%)	1.89 (+/- 2.32)

**Table 5:** Comparison of postnatal satisfaction scores between induction groups. Mean postnatal pages satisfaction scores with standard deviations for each overarching theme and independent t-test comparison of mean satisfaction scores between Misoprostol and Oxytocin study groups. In the open-ended pages assessment, some women mentioned delivery time, pain and anxiety, concepts that were directly questioned in the likert questionnaires. This allows direct comparison between the methodologies (Table 6). The mean satisfaction scores for the 3 questions were very similar in the two instruments, although only 13–24% of women identified the concepts of time to delivery, pain and anxiety as important in the pages questionnaire

	Overall mean satisfaction score (+/- SD)	Misoprostol group mean satisfaction score (+/- SD)	Oxytocin group mean satisfaction score (+/- SD)	Independent t-test		
				Mean difference	95% CI	*p*
Family	9.03 (+/- 1.60)	9.14 (+/- 1.66)	8.91 (+/- 1.54)	0.237	-0.22: 0.69	*0.152*
Looking to the future	7.25 (+/- 2.21)	7.31 (+/- 2.22)	7.03 (+/- 2.44)	0.284	-0.52: 0.62	***0.049***
Mother’s emotional wellbeing	7.72 (+/- 2.91)	7.45 (+/- 3.05)	7.94 (+/- 2.78)	-0.494	-1.20: 0.22	*0.086*
Mother’s physical health	5.19 (+/- 2.68)	5.77 (+/- 2.49)	4.68 (+/- 2.76)	1.087	-0.07: 2.24	***0.032***
Perspective on the baby	8.68 (+/- 2.02)	8.88 (+/- 1.80)	8.51 (+/- 2.20)	0.366	0.00: 0.73	***0.024***
Perspective on the birth	7.06 (+/- 2.94)	7.18 (+/- 2.85)	6.94 (+/- 3.02)	0.238	-0.14: 0.61	*0.105*

**Table 6:** Comparison of pages index importance and satisfaction scores with likert responses. The number of women citing each of these key concepts in the postpartum pages forms and the mean number of spending points (beans) allocated to their comments (note that concepts such as pain May be cited in various codes and if so, the values have been combined). Mean satisfaction scores for the codes or group of codes denoting each concept are provided and these can be directly compared to the mean likert scores for these areas

Satisfaction Questions (Likert scale)	PaGES Index				Satisfaction Score– Mean Likert score (+/- SD)
	Number (and %) of women citing each concept	Importance– mean number of beans allocated (+/- SD)	Concept satisfaction (in those who cited it)– mean score (+/- SD)		
Acceptability	No single code or group of codes represent this				7.44 (+/- 2.32)
Delivery time	*n* = 68 (13.1%)	3.13 (+/- 3.05)		6.66 (+/- 2.34)	6.11 (+/- 2.73)
Pain	*n* = 124 (23.9%)	2.32 (+/- 3.03)		4.82 (+/- 2.68)	3.63 (+/- 2.61)
Anxiety	*n* = 70 (13.5%)	1.81 (+/- 2.50)		4.44 (+/- 2.68)	4.58 (+/- 2.96)

### Integration analyses

**Fig. 4:**
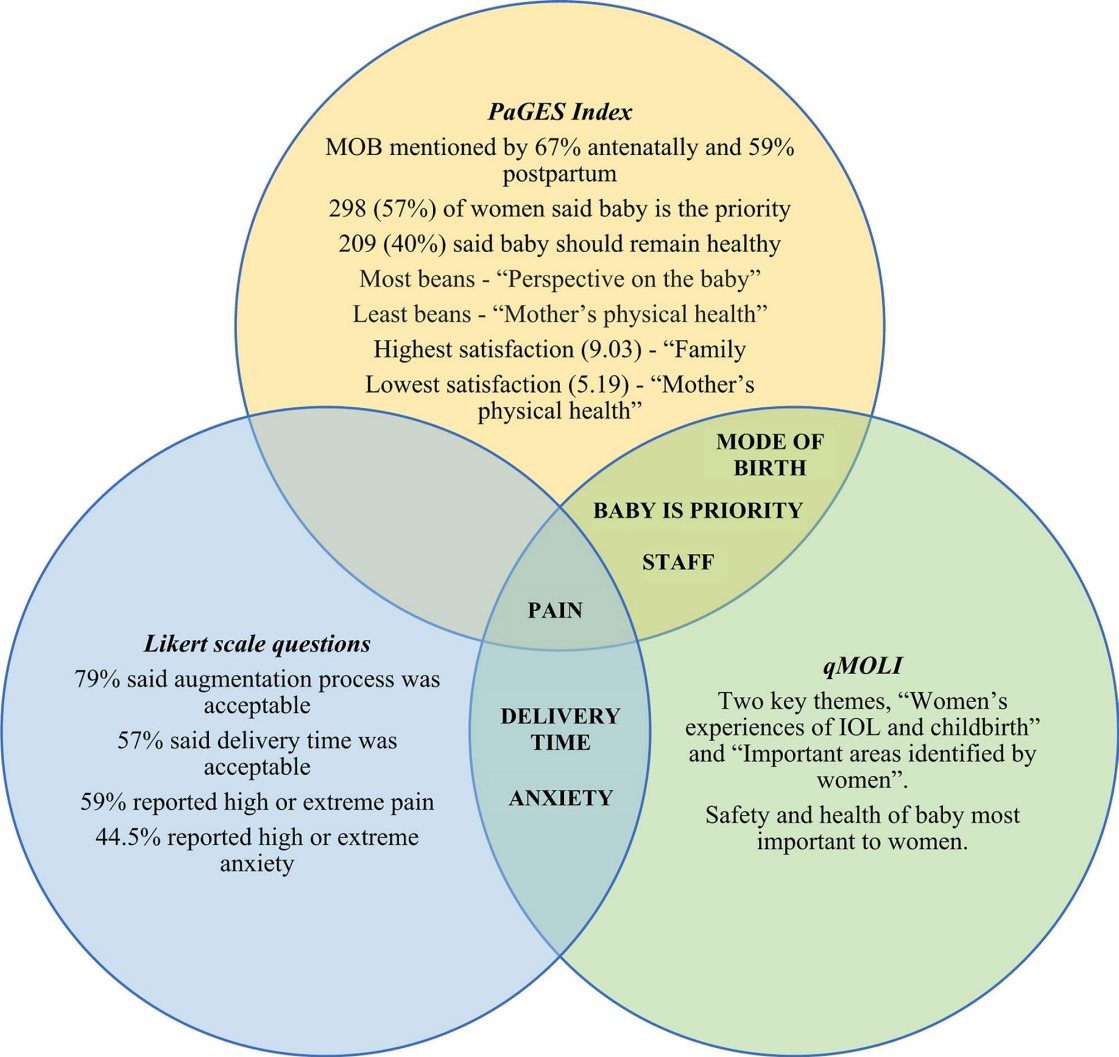
Integration of PaGES, Likert and qMOLI key findings. Venn diagram summarising the key findings of the three tools, demonstrating overlapping areas

A ‘contiguous approach’ to data integration was taken for this study [33]. Integration analyses were based on the postpartum data of 20 women who completed all three tools. Table 7 demonstrates three participants’ responses (see appendix 4 for full table). No association was found between mean Likert scores and overall postpartum PaGES scores, however common themes emerged from the PaGES Index, Likert and qMOLI data (Fig. 4).

Women who reported ‘high’ or ‘extreme’ Likert pain scores almost always discussed experiencing severe labour pain in their qMOLI interview. These women also generally gave a PaGES Index statement relating to pain with a low satisfaction score. For example, participant 1-1190 gave a Likert pain score of 4 (high) and reported that she “*had a lot of pain in the abdomen”.* She also provided a statement relating to pain on her postpartum PaGES form which had a low satisfaction score of 3. However, there were exceptions such as participant 1-1189 who also gave a Likert pain score of 4 but in her qMOLI interview said, *“I had problems with BP*,* otherwise I did not have much trouble with labour pains”*. This participant discussed delivery time in her qMOLI interview and PaGES form but did not provide a score on Likert- response form.

Women did not express opinions on either augmentation method unless prompted in qMOLI interviews and PaGES statements, but Likert augmentation scores demonstrated acceptable (49.8%) or very acceptable (28.7%) induction. Most women mentioned mode of birth in PaGES and qMOLI and every woman who expressed dissatisfaction with caesarean section on the postpartum PaGES form also discussed this in their qMOLI interview. For example, participant 2-0102 said *“In Caesar there is a problem”* and made a PaGES statement that coded for “Mode of birth (MOB) - dissatisfaction with CS”.

The PaGES index scores reveal how predetermined Likert scale questions can present false impressions. For two participants (IDs 1-1189 and 1-1190), the Likert scale responses suggest that the participant was unhappy with the birth process. This was based on questions related to time in labour, pain and anxiety. The PaGES Index however reveals that the women’s main priorities were not length of time in labour and anxiety, but their baby’s safety, the desire to have a vaginal birth and the baby’s gender, none of which were asked about in the Likert questions.

**Table 7:** Integration of results. Sample likert, pages and qMOLI responses from three of the 20 randomised participants who completed all three tools colour coded with negative (red), neutral (yellow) and positive (green) scores. Overall pages scores were calculated by totalling the multiplied postpartum bean and satisfaction scores from each participant’s postpartum statements. Scores for all 20 women are shown in appendix 4. *Abbreviations: MOB– mode of birth*,* NVD– normal vaginal delivery*

ID (qMOLI ID)	Group	Likert questions				PaGES Index				qMOLI	Interpretation
		Augmentation	Delivery time	Pain	Anxiety	PP statements	Beans	Satisfaction	Overall Score		
1-1190	Miso	2	3	4	4	Misc– medication	0	6	175	“My husband wanted a baby girl.” “Should deliver early, that’s only.” “I wanted normal (delivery).” “I thought there should not be more trouble. There was a lot of pain in the abdomen.”	High pain reported across all three tools
						Pain– general	2	3			
						Gender– important	5	9			
						MOB– satisfaction with NVD	3	8			
						Gender– family not important	0	7			
						Happy– to have baby	10	10			
1-1189	Oxy	2	-	4	5	Blood pressure	5	3	131	“I asked madam, how long will my delivery take.” “I wanted my baby to come out safely.” “I had a problem with BP otherwise I didn’t have that much trouble during labour pains.” “I am happy. Happy means my delivery occurred earlier.”	High pain on Likert form but says did not have much trouble with pain Delivery time discussed in qMOLI and PaGES, but no Likert score given
						Delivery– timeframe	0	4			
						Delivery– story of birth	2	1			
						Worried– baby	0	2			
						Gender– important	3	8			
						MOB– satisfaction with NVD	10	9			
2-0102	Miso	2	4	4	4	MOD - dissatisfaction with CS	5	5	118	“Baby should be good; mother should be healthy.” “Family members were too upset. Like, why it’s not happening yet.” “Nervousness is there na… How will it happen, what will happen? Whether it would be normal or not?” “That’s only that in normal delivery all remains normal. And in Caesar there is a problem.”	High Likert anxiety score also discussed in qMOLI interview PaGES says family happy/good but in qMOLI family described as upset Dissatisfaction with CS in PaGES and qMOLI
						Baby– healthy	7	7			
						Long-term future– general	1	1			
						Postpartum– now I am good	1	8			
						Family– happy/good	3	10			
						Misc– general	1	1			
						Family - happy/good	2	2			

Figure 4 summarises the key findings of the postpartum PaGES Index, qMOLI interviews and Likert questionnaires, and highlights overlapping themes between participants’ responses to the three tools. Areas of convergence between the PaGES statements and qMOLI quotes included mentions of staff, mode of birth and the baby’s health and wellbeing. Acceptability of delivery time and anxiety were assessed through the Likert questionnaire and were also discussed in qMOLI interviews. Pain was a common theme across all three tools.

## Discussion

In this clinical trial on labour induction in India, satisfaction was assessed using three methods and the data compared. Analysis of standard, closed Likert questions revealed that women were generally satisfied with the IOL process with most women reporting the process of augmentation was acceptable (79%), as well as the time it took to deliver (58%). Many, however, stated high levels of pain and anxiety (59% and 45%). Pain, anxiety and fear were also themes from the 20 semi-structured postpartum interviews, but women revealed that the most important concept for them was the safety and health of the baby, and that this took priority over induction method, mode of birth and mother’s own health. Other key areas from the interviews were blood pressure, birth experience, pain and the care given by staff. However, following childbirth, women were happy and pleased to have a baby. Like the interviews, the PaGES results found that having a healthy baby was the main priority for women before and after birth. In addition, women highlighted mode of birth as a priority and expressed a clear preference for vaginal birth. “Perspective on the birth” comments were allocated the highest proportion of beans whilst “mother’s physical health” statements received the fewest beans. Women expressed lowest satisfaction with “mother’s physical health” and highest satisfaction with “family”. Overall satisfaction in the MOLI study was high, however the PaGES index revealed that it was lower in the specific areas that the women raised as important. The PaGES satisfaction scores and Likert scores yielded similar results for delivery time, pain and anxiety, however of these, only pain was raised as one of the most important issues for women in the postpartum PaGES form.

A major strength of this study was the large study population and high response rate, which increased the validity of findings and allowed for meaningful comparison between the methodologies. However, the small number of interviews means that direct comparisons between the three methodologies was limited to the 20 women in the qualitative study. It was possible however to directly compare the Likert and PaGES results and to compare overall conclusions on satisfaction from each method.

**Table 8:** Comparison of data collection methods. An overview of the three data collection methods; likert scale questionnaires, qualitative interviews, and the pages Index - detailing their respective pros, cons, and recommendations for future studies

Method	Pros	Cons	Recommendations for researchers
Likert Scale Questionnaires	Quick and efficient Easy to understand Can be incorporated into large studies Allows for rapid statistical analysis	Closed questions may not reflect participant priorities Potential central tendency/response bias Limited by predetermined questions Inadequate for capturing overall satisfaction Limited number of questions	Consider using a 4-point Likert scale to reduce neutral responses Ensure questionnaires with multiple questions utilise the same numerical scale to facilitate statistical analysis Increase the number of questions for more comprehensive insights
Qualitative Interviews	In-depth exploration of participant experiences Rich qualitative data generated Captures a wide range of issues raised by participants	Time-consuming to conduct and analyse Limited sample size due to time constraints Difficult to generalize findings Transcription and translation challenges Skilled researchers needed	Use high quality transcription and translation services (role for AI in the future? ) Provide training for researchers in qualitative methods to improve data collection and analysis. Ensure adequate funding is requested for high quality alongside qualitative studies
PaGES Index	Allows for both quantitative and qualitative analysis Participant-generated data driven by participants’ priorities and focus Data can be obtained from all trial participants Can be used in large studies Can be incorporated into trial case report forms Provides more detailed insights than Likert scales	Data complexities (e.g., inaccurate bean counts, duplicated statements) Ambiguity in coding and categorisation Time-consuming post-data collection processes	Explore simplifying post-data collection processes (e.g., using predetermined code lists or limiting participants’ responses) Adapt the method for other settings based on initial testing

Each of the three data collection methods has strengths and weaknesses (Table 8). Likert scale questionnaires are quick, efficient and easy to understand measures of satisfaction that can easily be incorporated into large studies [34]. As quantitative results are collected on all women within the study, interpretation and statistical analysis are rapid in order to draw conclusions and identify trends. However, the closed questions are predetermined by the research team, and they do not, as seen clearly in this study, necessarily reflect the priorities of the participants. The research team’s choice of questions (on pain, anxiety, length of time in labour, and the acceptability of the augmentation method) reflect their perceptions of what might vary between the two arms of the study, not women’s overall satisfaction. To the casual reader it therefore gives a distorted view of women’s satisfaction. Furthermore, Likert scales have their own methodological challenges including central tendency bias [35], as participants often avoid using extreme response categories. This was observed in the present study. Response bias may also occur when patients do not feel comfortable expressing their honest response or are reluctant to express negative views to the questioner [36]. As only four Likert scale questions were included, we were only able to ascertain limited information regarding patient satisfaction. Another limitation was the lack of synonymity between Likert scales used. Two questions asked participants to rate the acceptability of their augmentation and delivery time on a scale ranging from very acceptable to very unacceptable, whilst the other two asked patients to rate their pain and anxiety on a scale of none to extreme. Thus, scores could not be assimilated to generate an overall score. Finally, we used a 5-point Likert scale. However, a 4-point scale may have been more effective as this would eliminate the possibility of selecting a neutral or moderate response [37].

Qualitative interviews are a standard method to collect in-depth data on the experiences of study participants. However, the time-consuming nature of both interviews and analysis limits the sample size to when ‘data saturation’ has been reached– that is when no new information is being revealed in interviews. This allows the research team to understand the breadth of issues raised by participants, but not their frequency. It is difficult therefore to use the results to generalise about the satisfaction of the participants– only to describe the range of their experiences. This is in direct contrast to the Likert and PaGES results where the opinion of every participant was reported, and frequency could be ascertained. There are additional limitations of the qualitative methodology, mainly centred around transcription and translation errors, though the team attempted to mitigate potential bias from inaccuracies through group discussion.

A major strength of using the PaGES Index in the present study was that all women could be sampled, which allowed for inter-group statistical comparison. The qualitative element of the PaGES Index allowed women to freely give their opinions, whilst the quantitative component facilitated statistical analysis. However, there remained some data complexities to deal with such as bean counts not adding up to 20 and duplicated statements, both of which resulted in minor inaccuracies. Furthermore, there was ambiguity in the coding of certain statements, for example where two potential codes would have been appropriate for one statement. In these instances, a code was allocated based on group consensus, but we found that both codes usually amalgamated into the same, higher level overarching theme regardless. Overall, the PaGES Index was an easier data collection method to incorporate into the trial than interviews, which had to be scheduled separately. It also provides a more detailed insight into women’s experiences and satisfaction than a purely quantitative method such as Likert questionnaires. Furthermore, though the PaGES Index was used in a birth setting in this study, this PROM could be adapted to other trials and study populations.

Post data-collection processes, including translating, data cleaning, coding and recoding, are very time consuming and complex. To further simplify the analysis, future studies might consider only implementing the PaGES Index post-intervention. Alternatively, researchers could limit the number of statements made by each participant to 5, which would still be sufficient to capture the participants’ views. It may also be more practical for participants to select codes from an extensive predetermined list once an initial PaGES dataset for a clinical situation and setting has been obtained and coded.

### Comparison with other studies

As the PaGES Index was pioneered in this study, there have been no previous published comparisons with other methodologies. Furthermore, virtually all previous qualitative research on birth outcomes, whether induced or not, comes from high income settings. It may seem surprising therefore that our satisfaction findings are consistent with much of the existing quantitative research of women’s experience of IOL. Generally, studies assessing satisfaction through Likert scale questions report overall positive birth experiences. A high-income setting study assessing maternal satisfaction with induction using a 10-point scale found that women in both the treatment and placebo groups expressed overall satisfaction following induction and birth [38]. Qualitative interviews with women however reveal many negative experiences, often due to receiving insufficient information and issues surrounding decision-making, support and environment [39]. Dissatisfaction with IOL is associated with a lack of knowledge [40], and women are often “surprised” to find they need to be induced and experience stress whilst awaiting active labour [41]. Researchers therefore conclude that women should be kept updated at each step of their induction process and healthcare professionals should ensure women are aware about the potential need for additional interventions and pain management [42]. Fear of childbirth before labour is a risk factor for a negative experience of IOL [43]. Many women reported high levels of pain following induction, a finding that is echoed by other qualitative studies [44, 45]. However, no systematic reviews have synthesised evidence relating to women’s experiences of pain with various induction methods. Women describing their experiences of IOL often mentioned care from staff, demonstrating that women undergoing IOL can have a positive birth experience if well supported [46].

### Implications

Considerable understanding of the priorities, experience and satisfaction of women in the MOLI study has been gained as a result of the triangulated analysis of the PaGES Index, Likert questionnaire and qMOLI interviews results. Each of the three methods has its own strength and a role to play in assessing birth satisfaction. Likert scales provide rapid data that is easy to assess and analyse and can be useful if research funds are limited. However, the results need to be viewed with caution as they only reflect the priorities of the research team and do not explore the range or relative importance of participants’ views. Qualitative data generated through semi-structured interviews is time consuming to collect and analyse, and can only tell of the breadth of experience, not their relative frequency. They cannot therefore be used to compare two randomised groups or to describe the frequency of the experiences. They do, however, effectively explore the depth of the participants’ experiences and help to explain the quantitative data. They are best used alongside quantitative analyses. The new PaGES Index seeks to provide the ‘best of both worlds’ with an initial rapid qualitative assessment using open questions, followed by a quantification of both the relative importance and satisfaction of each item generated.

## Conclusions

The PaGES Index, Likert questionnaires and semi-structured interviews provide varied data which are difficult to compare directly. The Likert questions only asked about four specific areas, but sample all randomised participants. Conversely, the interviews asked open questions about a range of topics but to a limited sample of women. The PaGES index is a hybrid measure which asks brief open questions about priorities to all women, then allows women to quantify their relative importance and satisfaction with each. This allows highly granular data to be collected and quantified from all participants. This study shows that the PaGES results align with both Likert and interview results. We therefore conclude that the PaGES Index is a feasible PROM for collecting detailed qualitative and quantitative insights. Future research should further validate the PaGES Index in other trials and study populations, including among groups receiving care for other health conditions.

## Electronic supplementary material

Below is the link to the electronic supplementary material.

Supplementary Material 1

Supplementary Material 2

## Data Availability

Until full results are published, only the study investigators will have access to the data files. Subsequently, the full database, including the PaGES Index data, will be made available to other researchers upon request. Efforts will also be made to use open access databases to ensure our data is widely accessible with minimal restrictions, adhering to MRC and Wellcome Trust guidelines.
