# Supplementary material for: Evaluating women’s experiences and satisfaction with labour induction in India: a comparison of the participant generated experience and satisfaction (PaGES) index with standard methods
Source: BMC Pregnancy Childbirth. 2025 May 28;25:619. doi: 10.1186/s12884-025-07731-9 (PMC12117871; doi:10.1186/s12884-025-07731-9)
Supplement: Supplementary file 1 — Supplementary Material 1 [file 12884_2025_7731_MOESM1_ESM.docx]

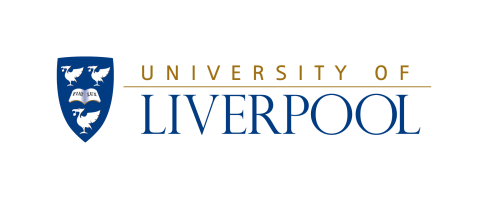

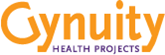

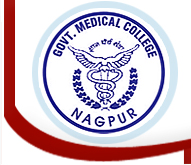


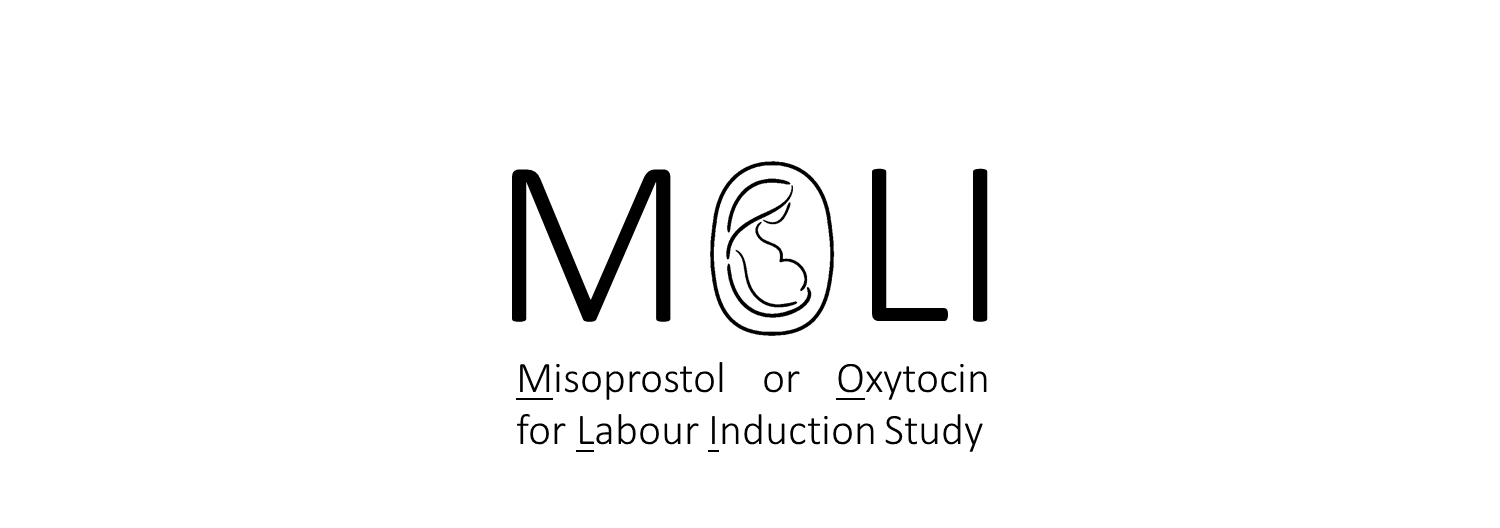


**An alongside qualitative study exploring patients’ and health care professionals’ expectations and experiences of labour induction with misoprostol and oxytocin for hypertension in pregnancy in India**

Trial Protocol

Chief Investigator: Professor Andrew Weeks

Trial Sponsor: University of Liverpool

Funder: MRC/DfID/Wellcome Trust Joint Global Health Trials Fund

| Trial Sponsor:  University of Liverpool  Research Support Office  Waterhouse Buildings  3 Brownlow Street  Liverpool L69 3GL  United Kingdom | EudraCT number:  CTA Reference Number:  ISRCTN:    Research Ethics Ref:  Sponsor Ref:  Funder’s Ref: MR/R006/1801 |
| --- | --- |

# Signature Page

The undersigned confirm that the following protocol has been agreed and accepted and that the Chief Investigator agrees to conduct the study in compliance with the approved protocol and will adhere to the principles outlined in the Declaration of Helsinki, the Sponsor’s SOPs, and other regulatory requirements.

I agree to ensure that the confidential information contained in this document will not be used for any other purpose other than the evaluation or conduct of the investigation without the prior written consent of the Sponsor.

I also confirm that I will make the findings of the study publicly available through publication or other dissemination tools without any unnecessary delay and that an honest accurate and transparent account of the study will be given; and that any discrepancies from the study as planned in this protocol will be explained.

| **For and on behalf of the Study Sponsor:** | | |
| --- | --- | --- |
| Signature:  .............................................................................................. |  | Date: ....../....../...... |
| Name (please print):  ............................................................................................. |  |  |
|  |  |  |
| **Mr Alex Astor**  University of Liverpool  Research Support Office  Waterhouse Buildings  3 Brownlow Street  Liverpool L69 3GL  **Chief Investigator:** | | |
| Signature: .............................................................................................. |  | Date: ....../....../...... |
| Name: (please print):  .............................................................................................. |  |  |

**Professor Andrew Weeks**

Professor of International Maternal Health

Sanyu Research Unit, Department of Women's and Children's Health

University of Liverpool

c/o University Department, First Floor

Liverpool Women's Hospital

Crown Street

Liverpool L8 7SS

# Full Study Title

An alongside qualitative study exploring patients’ and health care professionals’ expectations and experiences of labour induction with misoprostol and oxytocin for hypertension in pregnancy in India.

# Short Title

qMOLI - a qualitative assessment of Misoprostol or Oxytocin for Labour Induction.

# Glossary

| CRF | Case report form |
| --- | --- |
| CTG | Cardiotocograph |
| CTU | Clinical trials unit |
| DFID | Department for International Development |
| DMWH | Daga Memorial Women’s Hospital |
| FHR | Fetal heart rate |
| GCP | Good Clinical Practice |
| GHP | Gynuity Health Projects |
| GMC | General Medical College (Nagpur) |
| INFORM | Induction with FOley catheter OR Misoprostol (trial) |
| IOL | Induction of labour |
| IRB | Institutional Review Board |
| LMIC | Low/Middle Income Countries |
| LSTM | Liverpool School of Tropical Medicine |
| MGI | Mother Generated Index |
| M/M | Misoprostol/Misoprostol |
| M/Ox | Misoprostol/Oxytocin |
| MRC | Medical Research Council |
| RCT | Randomised controlled trial |
| REC | Research Ethics Committee |
| SAE | Serious adverse event |

# Contents Page

[Signature Page 2](#_Toc3227721)

[Full Study Title 3](#_Toc3227722)

[Short Title 3](#_Toc3227723)

[Glossary 3](#_Toc3227724)

[Contents Page 4](#_Toc3227725)

[Collaborators’ Team and Contact Details 6](#_Toc3227726)

[Protocol Summary 8](#_Toc3227727)

[Key Roles, Responsibilities and Relationships 9](#_Toc3227728)

[Funding 10](#_Toc3227729)

[Contact Details: Individuals 11](#_Toc3227730)

[MOLI Study Flow Diagram 12](#_Toc3227731)

[Background 12](#_Toc3227732)

[Rationale 14](#_Toc3227733)

[Research Question/Aims 15](#_Toc3227734)

[Specific Objectives 15](#_Toc3227735)

[Theoretical Framework and Methodology 15](#_Toc3227736)

[Design 16](#_Toc3227737)

[Study Setting 16](#_Toc3227738)

[Sampling 16](#_Toc3227739)

[Pre IOL Sampling Frame 17](#_Toc3227740)

[Post IOL Sampling Frame 17](#_Toc3227741)

[Eligibility Criteria 18](#_Toc3227742)

[MOLI RCT participants (women being induced) 18](#_Toc3227743)

[MOLI practitioners 18](#_Toc3227744)

[Recruitment 18](#_Toc3227745)

[Data Collection 19](#_Toc3227746)

[Analysis 20](#_Toc3227747)

[Consent 21](#_Toc3227748)

[Ethical and Regulatory Considerations 22](#_Toc3227749)

[Research Ethics Committee (REC) and Other Regulatory Review and Reports 23](#_Toc3227750)

[Regulatory Review and Compliance 23](#_Toc3227751)

[Amendments 23](#_Toc3227752)

[Peer Review 23](#_Toc3227753)

[Protocol Compliance 23](#_Toc3227754)

[Data Management, Protection and Patient Confidentiality 23](#_Toc3227755)

[Patient and Public Involvement 24](#_Toc3227756)

[Indemnity 24](#_Toc3227757)

[Access to the Final Study Dataset 25](#_Toc3227758)

[Discontinuation of Study 25](#_Toc3227759)

[Withdrawal of Consent 25](#_Toc3227760)

[Dissemination Policy 25](#_Toc3227761)

[Authorship Eligibility Guidelines 26](#_Toc3227762)

[References 26](#_Toc3227763)

[Appendices 28](#_Toc3227764)

[Appendix 1- Required Documentation 28](#_Toc3227765)

[Appendix 2 – Schedule of Procedures for q MOLI 29](#_Toc3227766)

[Appendix 3 – Schedule of Procedures for RCT 29](#_Toc3227767)

[Appendix 4 – Amendment History 30](#_Toc3227768)

[Appendix 5 – MOLI Interview Guide 1: Women, pre IOL 31](#_Toc3227769)

[Appendix 6 – MOLI Interview Guide 2: Women, postnatal 34](#_Toc3227770)

[Appendix 7 – MOLI Interview Guide 3: MOLI Interview Guide: Staff focus groups – before start of trial 37](#_Toc3227771)

[Appendix 8 - MOLI Interview Guide 4: Staff focus groups during trial 39](#_Toc3227772)

[Appendix 9 – Patient consent form 40](#_Toc3227773)

[Appendix 10 – Patient information leaflet 40](#_Toc3227774)

[Appendix 11 – MOLI Randomised Controlled Trial Protocol 40](#_Toc3227775)

# Collaborators’ Team and Contact Details

| Chief Investigator | | |
| --- | --- | --- |
| Professor Andrew Weeks (AW) | Professor of International Maternal Health,  University of Liverpool,  Liverpool Women’s Hospital,  Crown Street,  Liverpool, L8 7SS | Tel: +44-151-795 9578  [aweeks@liverpool.ac.uk](mailto:aweeks@liverpool.ac.uk) |
| Qualitative Lead | | |
| Dr Kate Lightly  (KL) | Clinical Research Fellow  University of Liverpool, Dept of Women’s and Children’s Health,  Liverpool Women’s Hospital,  Crown Street,  Liverpool, L8 7SS | Tel: +44-7875642837  [klightly@liverpool.ac.uk](mailto:klightly@liverpool.ac.uk) |
| Co-investigators | | |
| Dr Shuchita Mundle  (SM) | Professor, Department of Obstetrics & Gynaecology,  Government Medical College,  Nagpur, India 440003 | Tel: +91-9822706087  [srmundle@gmail.com](mailto:srmundle@gmail.com) |
| Dr Carol Kingdon | Senior Research Fellow  School of Community Health and Midwifery, Faculty of Health and  Wellbeing, University of Central Lancashire, Preston, UK | Tel: +44-151-7959823  [CKingdon@uclan.ac.uk](mailto:CKingdon@uclan.ac.uk) |
| Dr Hillary Bracken | Senior Director,  Gynuity Health Projects,  15 East 26^th^ Street, Suite 801  New York, NY 10010, USA | Tel: +001 212 448 1230  [hbracken@gynuity.org](mailto:hbracken@gynuity.org) |
| Professor Zarko Alfirevic | Professor of Fetal and Maternal Medicine,  Head of Women’s and Children’s Health,  University of Liverpool,  Liverpool Women’s Hospital,  Crown Street, Liverpool, L8 7SS, UK | Tel: +44-151-795 9550  [zarko@liverpool.ac.uk](mailto:zarko@liverpool.ac.uk) |
| Professor Beverly Winikoff | President,  Gynuity Health Projects,  15 East 26^th^ Street, Suite 801  New York, NY 10010, USA | Tel: +001 212 448 1230  [bwinikoff@gynuity.org](mailto:bwinikoff@gynuity.org) |

**Professor Andrew Weeks**, a clinical academic obstetrician from the University of Liverpool, will be the Chief Investigator. He will have the overall responsibility for trial design, conduct, analyses and reporting. He will make regular visits to the trial sites to ensure high quality practice but will delegate the day-to-day running to the experienced Nagpur-Gynuity team.

**Dr Kate Lightly**, a clinical research fellow and obstetrician from the University of Liverpool, is the lead for the qualitative MOLI work. She has written the protocol, will lead the focus groups, analyse the data and write up the findings.

**Dr Shuchita Mundle**, a Professor of Obstetrics and Gynaecology, from the Department of Obstetrics and Gynaecology, Government Medical College, Nagpur will be the Principal Investigator for India. Dr Mundle has extensive experience conducting clinical trials at this site and coordinating trials with partner institutions including Daga Women’s Hospital, Nagpur. She has a special interest in the management of pre-eclampsia in low-resource settings and has been principal investigator for several international trials with Gynuity Health Projects, including the successful INFORM trial [1].

**Dr Carol Kingdon**, a Senior Research Fellow, from the University of Central Lancashire, is a medical sociologist and experienced qualitative researcher.

She has multiple qualitative publications including in the Lancet. She had provided feedback on the protocol, will help to analyse the data and write up the findings.

**Dr Hillary Bracken** is an experienced qualitative and quantitative researcher, from Gynuity Health Projects. She will act as a trial manager in the US and manage the budget. Drs Mundle and Bracken have worked closely together in research in India for over 9 years.

**Professor Zarko Alfirevic** a clinical academic obstetrician and co-ordinating Editor of the Cochrane Pregnancy and Childbirth Group, Liverpool will assist in the design of the trial, development of the trial instruments and analysis of the trial results.

**Professor Beverly Winikoff** and Dr Hillary Bracken from Gynuity Health Projects will serve as co-investigators and collaborate on the management of the trial.

Dr Mundle will be assisted by consultant obstetricians: **Dr Maushmi Tadas** (GMC) and **Dr Seema Parvekar** (Daga). Nine research coordinators in Nagpur will assist with the data collection, routine trial management and data entry at the clinical sites for the MOLI RCT and initial recruitment for q MOLI.

Protocol Summary

| Full Title: | An alongside qualitative study exploring patients’ and health care professionals’ expectations and experiences of labour induction with misoprostol and oxytocin for hypertension in pregnancy in India. |
| --- | --- |
| Acronym: | qMOLI |
| **Study Design** | Qualitative study involving semi-structured interviews, with participants in a randomised trial, pre and post induction of labour and focus group discussions with healthcare professionals involved in the study. |
| **Study Participants** | 1.Pregnant women with hypertension/pre-eclampsia participating in the ‘Misoprostol or Oxytocin for Labour Induction’ (MOLI) study who consent to enter the qualitative study.  2.MOLI study staff. Practitioners who are involved in screening, recruiting, randomising and consenting participants to the MOLI study. |
| **Planned size of sample** | Semi-structured interviews (n=30-36) with MOLI participants pre and post IOL (conducted until data saturation – expected to be about 12 per group)   - 12 patients after recruitment to MOLI RCT but prior to the start of the induction process   2 MOLI RCT trial arm groups (some will have also been interviewed prior to induction)   - 12 women post IOL with misoprostol/misoprostol - 12 women post IOL with misoprostol/oxytocin   Staff focus group discussions (x10)  A focus group in each of the 2 recruitment sites before the start of the MOLI trial (n=2)  A focus group in each of the 2 recruitment sites and with each cadre of staff during the MOLI trial (n=8)   - Research assistants - Residents - Consultants - Midwives |
| **Planned study period** | Recruitment to the main MOLI trial is due to start once ethical approval is in place – estimated late 2019 and to continue for 24 months to late 2021.  Staff focus group discussions will be undertaken before the start of the trial in mid/late 2019. Patient semi-structured interviews with participants will be undertaken during the first few months of trial recruitment. Focus group discussions with staff will be repeated after the first 4-6 months of recruitment (end 2019/early 2020).  MOLI and q MOLI recruitment was halted due to the COVID pandemic. Therefore the study period will be extended until 06/2021. |
| **Research question/aims** | To assess the priorities, experiences and acceptability of induction of labour, for women being induced for hypertension in pregnancy in India, and clinician’s views on the feasibility, usability, acceptability and barriers to implementation of various induction protocols.   1. To explore patients’ perceptions, expectations, priorities, understanding of and concerns around induction of labour, prior to induction. 2. To explore the experiences, acceptability and satisfaction of patients post induction and any differences between the two RCT groups; misoprostol/misoprostol vs misoprostol/oxytocin regimes. 3. To better understand the feasibility, usability and acceptability of the different induction regimes to health care professionals. To explore potential barriers for implementing research findings into clinical practice and potential solutions. 4. To explore patients’ and staff perspectives of the fetal monitoring regimens during the induction process. |
| **Study Centres and Distribution:** | - Government Medical College, Nagpur, India. - Daga Memorial Women’s Hospital, Nagpur, India. |
| **Participant Study Duration:** | Maximum 90 minutes, depending on interview length |
| **Overall Study duration:** | 12 months |

# Key Roles, Responsibilities and Relationships

The MOLI Study management has been formalised with the main contract between the MRC/DfID/Wellcome Trust and the University of Liverpool. A Consortium Agreement has also been created between the University of Liverpool, the Liverpool School of Tropical Medicine (LSTM) and Gynuity Health Projects (GHP).

|  |
| --- |

Clinical Trials Unit (CTU): GHP will undertake the role of CTU in collaboration with the Chief Investigator, Professor Andrew Weeks, who will have overall management responsibility for the trial.

The University of Liverpool will undertake Sponsorship responsibilities in line with the UK Policy Framework on Health and Social Care Research (October 2017). It is recognised that the Sponsor has delegated certain tasks to the Chief Investigator and the CTU (GHP), as detailed in the Sponsorship Approval letter and the Permission to Proceed letter. The sponsor remains legally responsible for the trial.

# Funding

Funding for the MOLI RCT and q MOLI is provided by the MRC/DfID/Wellcome Trust Joint Global Health Trials Fund (ref MR/R006/1801) to support MOLI study-set up, delivery and reporting results for the 36 months.

| **Sponsor:** | **RCT Trial Management, Monitoring and Analysis:** |
| --- | --- |
| University of Liverpool  Research Support Office  University of Liverpool Waterhouse Buildings  3 Brownlow Street  Liverpool L69 3GL  United Kingdom  T: +44(0)151 794 8339  E-mail: [sponsor@liverpool.ac.uk](mailto:sponsor@liverpool.ac.uk) | Gynuity Health Projects  15 East 26th Street, Suite 801 New York, NY 10010  United States of America  Phone: 1 (212) 448-1230 Fax: 1 (212) 448-1260  E-mail@ [hbracken@gynuity.org](mailto:hbracken@gynuity.org) |

| Contact Details: Individuals | |
| --- | --- |
| **Individual Authorised to Sign the Protocol and Protocol Amendments on behalf of the Sponsor:** | **Chief Investigator (CI):** |
| Alex Astor*  University of Liverpool  Research Support Office  University of Liverpool Waterhouse Buildings  3 Brownlow Street  Liverpool L69 3GL  United Kingdom  T: +44(0)151 794 8339  E-mail: [sponsor@liverpool.ac.uk](mailto:sponsor@liverpool.ac.uk) | Professor Andrew Weeks  Professor of International Maternal Health  Sanyu Research Unit, Department of Women's and Children's Health  University of Liverpool  c/o Liverpool Women's Hospital  Crown Street  Liverpool L8 7SS  T: +44(0)151 795 9578  F: +44(0)151 795 9553  E-mail: [aweeks@liverpool.ac.uk](mailto:aweeks@liverpool.ac.uk) |
| **Principal Investigator (India)** | **Qualitative Lead** |
| Dr Shuchita Mundle,  Associate Professor / Consultant  Department of Obstetrics & Gynaecology  Government Medical College,  Nagpur,  India 440003  Tel: +91-9822706087  [srmundle@gmail.com](mailto:srmundle@gmail.com) | Dr Kate Lightly  Clinical Research Fellow  Sanyu Research Unit, Department of Women's and Children's Health  University of Liverpool  c/o Liverpool Women's Hospital  Crown Street  Liverpool L8 7SS  T: +44(0) 7875642837  E-mail: [klightly@liverpool.ac.uk](mailto:klightly@liverpool.ac.uk) |

# MOLI Study Flow Diagram


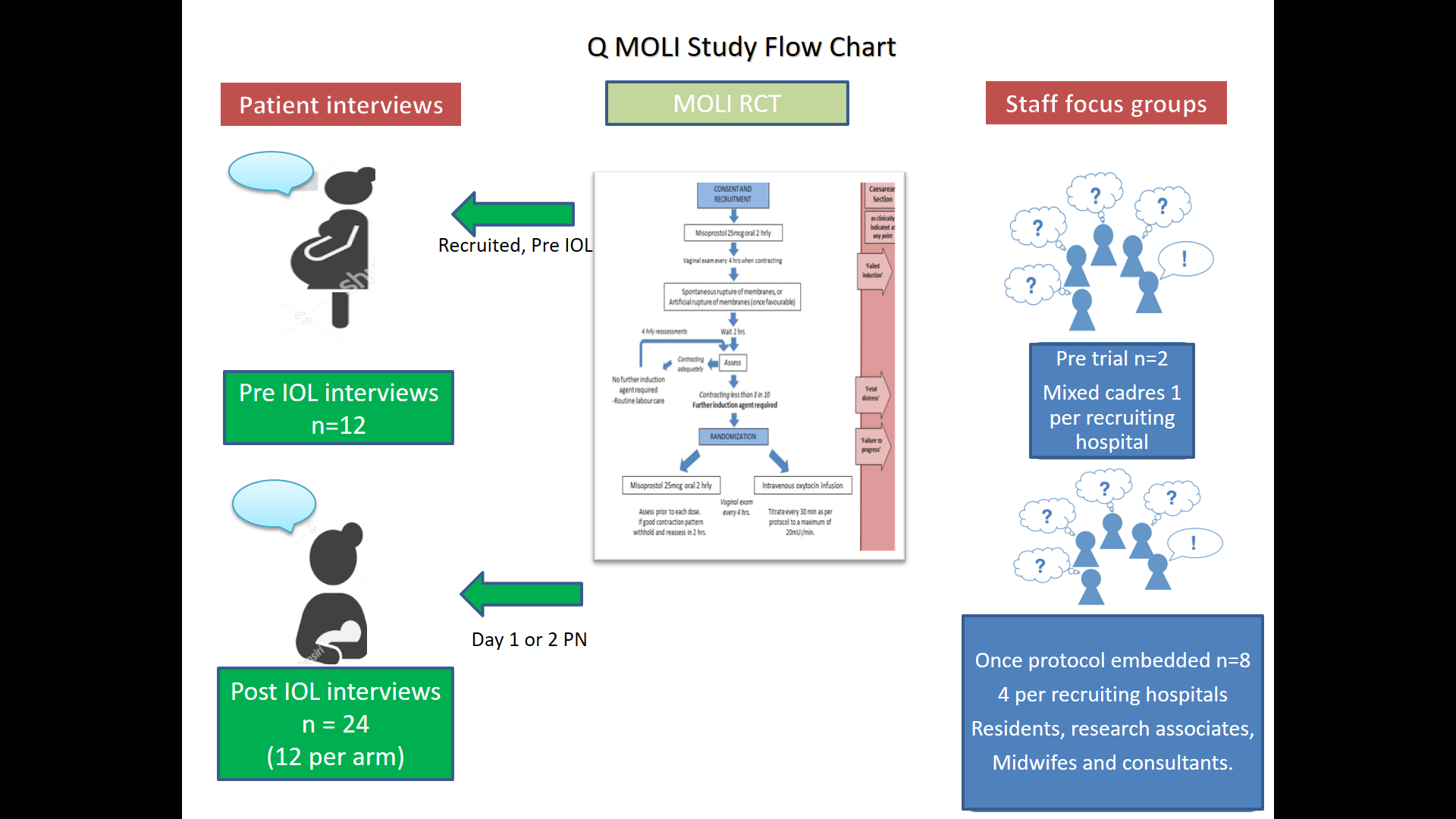


# Background

**For details of the MOLI randomised controlled trial, please see the MOLI trial protocol (appendix 11). This protocol constitutes the qualitative study only.**

Hypertensive disease in pregnancy is a major cause of the 300,000 maternal deaths that occur every year [2]. In South Asia alone, it is responsible for 10,000 deaths annually [3]. Much of this burden could be prevented by timely and effective delivery. Labour induction can be however, a difficult and potentially dangerous procedure for both mother and baby.

Rates of induction of labour are increasing around the world, up to 12.9% in India [4]. As the threshold for recommending induction decreases, the emphasis on safe and acceptable induction protocols, is becoming even more important. Standard practice for induction of labour is to use a prostaglandin (e.g. misoprostol) to ripen the cervix, then the amniotic membranes are broken. If the cervix is not dilated or there are no regular contractions, an intravenous infusion of oxytocin for uterine stimulation is often required [5]. The continuous oxytocin infusion involves attachment to an IV infusion for the duration of labour, which can result in medicalisation of labour and reduced mobility.

Misoprostol, a prostaglandin E1 analogue, given in low doses orally is a highly effective method for labour induction in low and middle income (LMIC) settings. Oral administration of 25 micrograms every 2 hours received a strong recommendation by WHO [6]. High quality 25mcg misoprostol tablets registered for labour induction have now become available on the market in India (Cipla Ltd) and Northern Europe (Azanta A/S), removing many of the previous logistical barriers to its use. An induction protocol continuing oral misoprostol into labour has never been directly compared to the standard regimen (of IV oxytocin) in a clinical trial. A randomised controlled trial of the two alternatives is therefore planned. Qualitative assessment alongside this RCT will determine how acceptability of IOL for patients is affected.

The ‘Misoprostol or Oxytocin for Induction’ (MOLI) trial is a pragmatic, open-label, randomised trial to compare a misoprostol/misoprostol labour induction protocol with the standard misoprostol/oxytocin protocol. We will recruit approximately 1000 women with hypertensive disease of pregnancy over 24 months in Indian government hospitals in Nagpur, India, in order to randomise 520 to the two RCT treatment arms. All participants will receive oral misoprostol 25mcg 2 hourly for cervical preparation (the first stage of induction). If there is a requirement for a further induction agent after the membranes have ruptured, then the patient will be randomized to receive further oral misoprostol (25mcg 2 hourly) or titrated intravenous oxytocin (standard treatment). The primary outcome for the RCT will be caesarean birth. Secondary outcomes will assess the efficacy of the induction process, maternal and fetal/neonatal complications and the relative cost-effectiveness. Fetal monitoring will be conducted using intermittent auscultation, with electronic fetal monitoring (EFM) in case of abnormalities. EFM has not previously been widely available at the study sites and this is being introduced in 2019. This study therefore provides an opportunity to also assess the staff and patients attitudes to intrapartum fetal monitoring techniques. The primary outcome for the RCT will be caesarean birth. Secondary outcomes will assess the efficacy of the induction process, maternal and fetal/neonatal complications and the relative cost-effectiveness.

This qualitative study will be run alongside the main MOLI RCT in order to explore women’s and health care professional’s experiences and perceptions regarding induction of labour and the two methods compared as part of the RCT. This will be done through semi-structured interviews and focus group discussions.

Qualitative research alongside the quantitative study will provide an in-depth assessment of the study phenomenon [7] both from patients’ and staff perspectives. This will improve external validity, interpretation of results, hearing the individual patients’ voices and potentially cost savings [8].

A recently published systematic review confirms the increasing amount of qualitative healthcare research in India over the last 20 years, with 246 papers being reviewed over 20 years [9]. A small number of qualitative studies have been undertaken with Indian pregnant or postnatal women exploring their preferences or experiences of birth [10]–[13]. We have not identified any that have explored induction of labour or intrapartum fetal monitoring and Indian women’s views, or priorities and perceptions surrounding this. There are also very few studies about women’s perspectives on fetal monitoring internationally, one undertaken in Tanzania highlighted that women felt they received better care whilst using a “Moyo” device for intrapartum fetal monitoring [14] .

Two recently published systematic reviews about patients’ perspectives of IOL highlight the small numbers of studies involved (8, 10.) All were undertaken in high income settings including UK, Ireland, Australia and Canada. The key themes for postdates IOL patients were the change in expectations due to lack of spontaneous onset of labour, the experience of “non decision” regarding choices and experiencing the induction process, as a series of steps to be undertaken within set timeframes [15]. The other review highlights what a difficult process this can be for women and the difference between expectation and actual experiences. Key themes were the ways in which decisions were made, the ownership of the process, the social needs of the woman and physical place of IOL [16]. The only study undertaken in LMIC was a cross-sectional questionnaire based interview in Nigeria about cervical ripening and induction of labour-awareness, knowledge and perceptions in antenatal attendees [17], [18].

This study will provide valuable information about Indian women’s views and priorities about IOL, highlight differences between expectations and lived experiences and provide a holistic assessment of use of oxytocin vs misoprostol for IOL from both staff and patients perspectives.

# Rationale

Induction of labour can be life saving for mothers and babies across the world for a huge variety of conditions. Working out the safest and most cost-effective way of doing this is vital. However, in an era of ever-increasing induction rates, induction protocols must also be acceptable to patients and incorporate their wishes and priorities where possible.

Running this qualitative study alongside the main RCT allows all aspects of this intervention to be assessed in detail. It means a richer understanding of the results of the RCT and their generalisability to other settings. The focus groups conducted with staff prior to the study will offer insights into their perceptions of issues and benefits of current techniques, without bias provided through the clinical study training.

It is well documented that it can take many years to implement research findings into clinical practice. Talking to health care staff about their own ideas, experiences and concerns will improve the understanding of potential barriers to implementing research findings into practice.

A recent Cochrane review [19], and much of the literature about IOL reports that more work needs to be done to explore women’s experiences of IOL. This has been partly done through two recent systematic reviews, however minimal work has been done in this area in LMIC. Better understanding of women’s views, ideas and priorities will allow health care professionals to take these into consideration for their own practice and advocate for their patients [19].

This work will contribute to understanding and implementation of respectful care in labour. Principles of respectful care include treating every woman with respect, providing her with information about what she might expect, asking her about her expectations, and involving her in the decisions about her care [20]. This study will help academics and clinicians to understand patients’ perspectives.

# Research Question/Aims

To assess the priorities, experiences and acceptability of induction of labour for women being induced for hypertension in pregnancy in India, and clinician’s views on the feasibility, usability, acceptability and barriers to implementation of various induction protocols.

# Specific Objectives

1. To explore patients’ perceptions, expectations, priorities, understanding of and concerns around induction of labour, prior to induction.
2. To explore the experiences, acceptability and satisfaction of patients post induction and any differences between the two RCT groups; misoprostol/misoprostol vs misoprostol/oxytocin regimes.
3. To better understand the feasibility, usability and acceptability of the different induction regimes to health care professionals. To explore potential barriers for implementing research findings into clinical practice and potential solutions.
4. To explore patients’ and staff perspectives of the fetal monitoring regimens during the induction process.

# Theoretical Framework and Methodology

The research team have a post positivism metatheoretical stance, which accepts the benefits of both quantitative and qualitative research and reflects the academic and clinical background of the team.

This research aims to improve the understanding of the patient’s feelings and real-life experiences of induction of labour, as well as patients and staff’s perspectives on the two arms of the RCT. Therefore, a qualitative research approach using the general principles of grounded theory will be adopted into the protocol. Grounded theory allows simultaneous collection and analysis of data whilst discovering emerging patterns in the data, and the generation of theories from this [21]. Using this methodology allows flexibility, as the number of participants is open, and can change if there are new findings. By immersing themselves in the data the researchers will explore what is important to patients and staff. The approach may be enhanced by the discovery of new information which may be used to amend the interview schedule and improve the understanding of participant experience.

Thematic analysis using the framework approach was developed in the late 1980’s and is now used widely in several areas including health research. It will allow comparison of the different experiences of protocols for induction of labour and identify commonalities and differences, before focusing on relationships between different parts of the data. The aim is to draw descriptive and/or explanatory conclusions clustered around themes [22]. The themes will be generated through an inductive approach, as no published research is available on Indian women’s perceptions of IOL on which to base a deductive approach.

We accept that the values, knowledge and background of the researchers will influence what is observed. We will acknowledge through reflexive accounting, the effect that our individual potential biases will have [23].

# Design

This study is a qualitative sub study of the MOLI randomised control trial.

# Study Setting

The MOLI RCT and qualitative study will run in two public hospitals in Nagpur, India – Government Medical College (GMC) and Daga Memorial Women’s Hospital (DMWH). Both hospitals have experience in the conduct of clinical trials, especially among this patient population. They encounter challenges associated with LMIC settings but are also sites of efficient and well-run university departments. This study is based on centres used for the INFORM study [1]. Dr Shuchita Mundle, based at GMC, is one of the co-investigators for this study and works closely with obstetric staff at DMWH.

Interviews with women will take place in a quiet and private space, close to the induction area. Most women speak Hindi or Marathi and so interviews will be conducted with an interviewer in the patient’s own language. Focus groups with staff will take place in a room within the department that is appropriately sized, quiet and away from the clinical area, whilst the staff are not responsible for any clinical duties.

# Sampling

The sample groups for patients will consist of three different groups of participants in order to get a holistic view: one before and two after the induction process. The two post IOL groups will explore the different arms of the RCT (misoprostol/misoprostol and misoprostol/oxytocin). Some women will be interviewed both before and after induction of labour, whereas others will just be interviewed afterwards. This will be done according to the sampling frame, availability of qMOLI team and willingness of participants to undertake a second interview. This will expand the potential for understanding concerns pre and post IOL and exploring expectations and experiences. The number of interviews required to understand the experience for participants is flexible and elements of the interview construct may evolve. It is expected that relatively large numbers of participants may need to be interviewed in order to get a clear understanding of their experiences, but we estimate that 12 in each group will be required. It is suggested that when following the principles of qualitative research, the sample size should follow the concept of saturation [21]. Data saturation will determine the appropriate time to discontinue interviewing. It is the point at which the collection of new data sheds no further light on the topic issue [24], [25].

Theoretical sampling will be used to ensure a diverse range of experience is collected. In the **pre IOL interviews** it will be important to include women who have previously had children, those that have not, and participants across the sites. In the in-depth **postnatal interviews**, the sample matrix will set out a minimal quota of: 6 who received an oxytocin infusion; 6 who received misoprostol after their membranes were ruptured; 6 delivered by caesarean birth; 6 who had vaginal deliveries and 6 who had a pre-IOL interview. It is appropriate to ensure specific numbers of patients as a minimum in each group, because it is necessary to fully explore perspectives from each RCT arm. Previous studies suggest that patient’s feedback on IOL/delivery is related to the outcome, so it is important to try to include a range of outcomes. We also aim to interview some women once and some twice, to assess different perspectives pre and post IOL. More women will be interviewed postnatally than antenatally, as we are studying the two RCT arms. The decisions around which women to interview once or twice will be dependent primarily on the patient wishes and availability of research staff.

# Pre IOL Sampling Frame

| **Criteria** | **Patients** |
| --- | --- |
| Primigravida | 3+ |
| Multigravida | 3+ |
| Social strata | Mixed |
| Hospital site | 2+ from each recruiting site |

# Post IOL Sampling Frame

| **Criteria** | **Patients** |
| --- | --- |
| Misoprostol/misoprostol regime - Vaginal delivery | 3+ |
| Misoprostol/misoprostol regime - Caesarean delivery | 3+ |
| Misoprostol/oxytocin regime – Vaginal delivery | 3+ |
| Misoprostol/oxytocin regime - Caesarean section | 3+ |
| Repeat interview of those already interviewed pre IOL | 6+ |
| Social strata | Mixed |
| Hospital site | 3+ from each recruiting site |

Focus group discussions with staff will occur prior to the beginning of the MOLI study and after 4-6 months of the trial starting, once the protocols are familiar to staff. All staff involved in the trial will be invited to participate, across the sites. Before the start of the trial the focus groups will take place in each of the recruiting sites, across all cadres of staff. The later focus groups will be undertaken with each staff cadre in each site (consultants, residents, research assistants and nursing/midwifery staff). Post-trial staff will be interviewed with their own peers to encourage confidence to share their views. A total of ten focus group interviews will be performed.

# Eligibility Criteria

## MOLI RCT participants (women being induced)

**Inclusion criteria**

- Women who are recruited to the MOLI RCT and are either antenatal, or post-induction and meet all of the eligibility criteria:
- Women who consent to join the qualitative study

**Exclusion criteria**

- Women who are not recruited to the MOLI RCT
- Women who lack the capacity to make an informed decision
- Women under the age of 16
- Women who have had a stillbirth in this pregnancy
- Women who are distressed/in pain
- Women too unwell to take part in interviews, or who need urgent intervention (in less than 2 hours)
- Where delay in starting the IOL process due to time of interview could cause harm to the patient
- Women who do not give consent to be in the study

## MOLI practitioners

**Inclusion criteria**

- Practitioners who are involved in screening, recruiting, randomising and consenting participants to MOLI RCT

**Exclusion criteria**

- Staff who do not wish to be included
- Staff who do not give their consent to participate

# Recruitment

**Patients** - Initial contact with potential qMOLI participants will be made by the RCT recruitment team. After recruitment to the MOLI RCT, women will be asked whether they would like to speak to a qualitative researcher about this additional aspect of the research.

If the MOLI recruit is willing to speak to the qMOLI researcher, then a research assistant will attend and discuss the qMOLI study. As well as verbal information, patients will also receive a patient information sheet in Hindi, Marathi or English and asked if they would like to participate. The PIS will be translated and back translated into Hindi and Marathi, in order to check accuracy. As with the main MOLI study, there is limited time for patients to consider entering the qMOLI study. This will vary according to the clinical scenario and urgency of IOL, but one hour is likely to be the maximum. Women who are unsure will therefore not be recruited to the study. If the patient is agreeable, recruitment and written consent will take place, in the patients’ own language. Separate written consent for qMOLI is necessary (see appendix), as it is a separate study to the MOLI RCT. Many more women will take part in the RCT than the qualitative study. Further opportunities to ask questions will be offered throughout this process.

**Staff -** The focus group discussions will be advertised, and information leaflets will be distributed so staff will have time to consider if they wish to participate. At the initial MOLI RCT training, the focus group element part of the study will be explained to staff. Those who register interest will be given a practitioner information sheet including details of topics that will be discussed in the focus group, duration of the group and location for the group. If a staff member decides to participate they will be asked to sign a consent form. They will also receive a letter after the focus group thanking them for their participation.

# Data Collection

Prior to the data collection, willingness to consent will be re-visited. It will be emphasised that the participant is free to withdraw at any time, without giving a reason, and that this will not affect them in any other way. Anonymity and confidentiality will be maintained, and any questions answered.

All interviews and focus groups will be digitally recorded on password protected recorders, with consent of the participants. Subsequent management of the data is described below in “data management, protection and patient confidentiality.”

**Patients** – Data collection with patients will all take place by semi-structured interview. Interviews will be conducted face-to-face, in the language of the patients’ choice (usually Hindi/Marathi), with the q MOLI team. The initial interview will occur after recruitment and consent to the MOLI RCT but before any cervical ripening drugs are administered, and then again after the birth, for some on the postnatal wards, usually on day 1-3 before the patient is discharged. Some will only be interviewed ante or post natally. This will be done at a time that is convenient for the patient, when both mother and baby are settled, and the clinical staff looking after the patient feel an interview is clinically appropriate. The q MOLI team is separate from the day to day aspects of the MOLI RCT and will not be involved in administering drugs or clinical care of patients.

A topic guide will be used to guide discussion and ensure that key topics are covered. The semi-structured interviews will be loosely based on open-ended questions, enabling pre-specified topics to be discussed and explored in detail, as well as new areas to be uncovered. This is the interview format most commonly used in healthcare research, as it provides participants with some guidance on areas to discuss which they may find helpful. It allows flexibility to discover and elaborate on information which may not have appeared pertinent to the researcher previously as well as providing focus on the topic, which will allow interpretation of the potential differences and similarities between the treatment arms of the group.

The interview guides were developed by:

- KL and the MOLI team after discussion with research assistants, (who previously worked on the INFORM study and will work on the MOLI RCT) as they are the staff group who spend the most time with patients and have the best understanding of patients’ perceptions,
- colleagues experienced in qualitative interviews in pregnant and postpartum women and
- colleagues who have experience of working with pregnant women in India.

**Staff** – Focus group discussion will be conducted in English, using interview guides to ensure that all topics are covered but allow flexibility to explore participants views. The interview guides are attached in the appendix of this protocol.

# Analysis

The framework analysis approach uses stages for analysis [26].

**Stage 1 – Transcription**

A verbatim (word for word) transcription of the interview will be taken from the audio recording. The transcripts will be uploaded onto NVIVO 12 software and then checked by researchers who conducted the interview. Transcripts will have large margins and adequate line spacing for appropriate coding and marking notes.

**Stage 2 – Familiarisation with the interview**

Both researchers will review the transcript and audio recording and ensure any contextual/reflective notes are included.

**Stage 3 – Coding**

This is an inductive evaluation, so open coding will be used to label anything which might be relevant. Codes could represent things, values, emotions and more impressionistic/methodological elements. This coding classifies the data, so it can be compared with the whole data set systematically. Each line will be reviewed individually looking for codes. At least the first few transcripts from each group will be independently coded by both researchers. Any differences will be discussed, and a consensus reached.

**Stage 4 – Developing a working analytical framework**

After coding the first few transcripts and each group of transcripts the researchers will meet to compare labels and agree on a set of codes to apply to the other transcripts. Codes will be grouped into categories, which are then clearly defined. Several iterations of this will be performed until no further codes emerge. An “other” code will be used to avoid missing any data.

**Stage 5 – Applying the analytical framework**

This working framework is then applied by indexing existing categories and codes. Each code will be assigned a number using the N-VIVO software.

**Stage 6 – Charting data into the framework matrix**

A spread sheet will be used to chart the data into a matrix via N-VIVO. The chart will include links to interesting or illustrative quotes.

**Stage 7 – Interpreting the data**

Characteristics and differences in the data will be identified, the researchers will then aim to map these and explore relationships and causality.

The data from each group of participants will be analysed separately and then merged.

Rigor and transparency will be maintained throughout the whole process by the research team conducting regular meetings to discuss the data.

# Consent

If the patient is agreeable to considering entry into the qualitative study (after recruiting and consenting to join the MOLI RCT), the research assistant will give them a patient information sheet in her own language to read. She will be given the opportunity to discuss her possible participation with her relatives, or friends or their legal representative or with staff experienced in obtaining informed consent. Then the q MOLI team will approach them on the ward.

The qualitative team will fully explain the study and answer any questions, whilst adhering to the principles of GCP, which have their origin in the Declaration of Helsinki.

Discussion will include:

- conversation between the potential participant or her legally acceptable representative and an individual knowledgeable about the research, about the nature and objectives of the study and possible risks associated with their participation, time commitments and data handling/access
- the presentation of written material (information sheet and consent documents)
- the opportunity for potential participants to ask questions
- assessment of capacity. A capable person will:
  - understand the purpose and nature of the research
  - understand what the research involves, its benefits (or lack of benefits), risks and burdens and ask appropriate questions
  - understand the alternatives to taking part
  - be able to retain the information long enough to make an effective decision.
  - be able to make a free choice
  - be capable of making this particular decision at the time it needs to be made
  - it is important that the patients understand that their interests will be protected and that they are not being coerced.

If the woman decides to participate then she will be asked to sign a consent form. In the event that a woman is not literate, the patient consent form (PCF) and information sheet will be read to her by the researcher. Upon reviewing the document, the researcher will explain the research study to the patient. This information will emphasise that participation in the trial is voluntary and that the participant may withdraw from the trial at any time and for any reason, without patient care being affected. All participants will be given an opportunity to ask any questions that may arise, have the opportunity to discuss the study with their friends/family member/doctor and time to consider the information prior to agreeing to participate. A contact point where further information about the trial may be obtained will be provided. Some Indian women may not be able to sign their own name but would be willing to place a finger/thumb print on the PCF. The PCF will be printed in triplicate; the original copy will be filed in the site office in the site file, a copy of the PCF will be given to the patient for their records and another copy in the patient’s notes.  Separate consent forms will be used for the MOLI RCT and qualitative study. The q MOLI documentation will be kept in a separate file, in a separate locked cabinet, in the MOLI site offices, than the main MOLI documentation.

# Ethical and Regulatory Considerations

A full suite of data management plans and SOPs have been written, audited and approved for the MOLI RCT by the trial sponsor, the University of Liverpool and these will be used, where relevant for the qualitative study.

The study will be conducted in accordance with

- The World Medical Association Declaration of Helsinki (1996)
- GHP Standard Operating Procedures
- International Conference of Harmonisation Good Clinical Practice (ICH GCP)

The protocol content is structured in line with the Health Research Authority protocol template.

If any issues are disclosed during the interviews that could cause any physical harm for the patient or her child, they will be discussed with the on-call doctor team and, if possible, the patient’s own consultant who would advise on how to manage these matters.

# Research Ethics Committee (REC) and Other Regulatory Review and Reports

Ethical approval will be sought from each of the recruiting sites in Nagpur and then the University of Liverpool. The MOLI RCT will already have been considered by the same ethics committees. The two studies are being submitted separately in order to start the RCT recruitment as soon as possible and because this study focuses on evaluation of both RCT protocols, as well as an overview of women being induced for hypertension in pregnancy in Nagpur’s wider perspectives on induction of labour.

# Regulatory Review and Compliance

Before any site can enrol patients into the study the University of Liverpool and the Chief Investigator will ensure that appropriate approvals from participating organisations are in place.

# Amendments

For any amendment to the study protocol the MOLI team will follow the MOLI protocol amendments SOP.

# Peer Review

The trial protocol will be reviewed by the MOLI Trial Steering Committee (TSC) which comprises of three independent experts and one patient representative, as well as an Indian expert in qualitative research. The MOLI proposal was peer reviewed at the grant application stage.

# Protocol Compliance

Protocol compliance will be managed via the MOLI TSC and TMG via the suite of relevant MOLI SOPS.

# Data Management, Protection and Patient Confidentiality

Data protection and patient confidentiality will be managed via the TSC and TMG via the suite of relevant MOLI SOPS.

Recordings will be made on two password protected digital recorders to guard against equipment failure. These will be anonymised, and attempts will be made to avoid using any identifiable data during the interview. Each participant will be given a code. These recordings will be stored securely within GMC’s research office. Completed audio-recordings will be transcribed and sent in password encrypted files. The recordings and transcriptions will be correctly stored on password protected files on the University computer in accordance with the UK General Data Protection Regulations (GDPR) 2018. Data in other forms e.g. N-VIVO, tree diagrams and excel spreadsheets will all be anonymised and stored in encrypted files, on password protected computers. The data in Nagpur, will be secured on a password protected computer, in a locked room and then also uploaded onto the M drive at the University of Liverpool, so as to ensure a second copy of the data is saved securely. The minimum number of individuals necessary will have access to this data, for quality control, audit and analysis. Any data transferred to co-investigators or sponsors will be anonymised. The data will be stored for 10 years as requested in the University of Liverpool SOPs on record retention. The data custodian will be the sponsor, the University of Liverpool.

All investigators and study site staff must comply with the requirements of the U.K GDPR 2018.

# Patient and Public Involvement

During the study development of the MOLI RCT, a scoping exercise was carried out to assess the potential for the MOLI trial. 14 doctors and 23 women who had undergone induction of labour were interviewed at Government Medical College. Most of the doctors (12/14) and women (22/23) would welcome a change to a misoprostol/misoprostol regimen, but only if it did not increase the risk of CS.

Ms Uma Sharma, a local consumer representative, who sits on the MOLI Project Steering Committee will review this protocol and interview guides and provide information from a patient perspective*.* She will provide feedback which will be incorporated at the planning stage of this project.

The interview guides have been discussed at length with research assistants (as they spent the most time with patients during the previous INFORM study) and clinicians that have previously worked in Nagpur.

# Indemnity

There are no specific indemnity arrangements for this qualitative study, other than the standard local hospital arrangements. KL will be personally insured against litigation during her time in India. The MOLI RCT has its own indemnity cover (see MOLI protocol for full details).

# Access to the Final Study Dataset

The Chief Investigator, Principal Investigator (s), qualitative lead and other qualitative researchers will have access to the full dataset.

# Discontinuation of Study

This study will end on completion of analysis of the data from the last interview of the study (whether patient interview or staff focus group).

In the event that the study is discontinued by the MOLI RCT TSC, participating sites will be advised on any actions that are required with regards to individual participants follow-up. Participants will otherwise be treated according to the usual standard clinical care.

# Withdrawal of Consent

The participant/legal representative is free to withdraw consent at any time without providing a reason and without any detriment to care. The rights and welfare of the participant will be protected by emphasising to them throughout the trial that the quality of medical care will not be adversely affected if they decline to participate or discontinue participation in the trial.

Generally, follow-up will continue unless the participant/legal representative explicitly withdraws consent for follow-up.

Where the participant wishes to withdraw consent, there will be clarification whether this is withdrawal of consent for data already collected or for future data collection. Centres should explain the importance of remaining in trial follow-up and of the importance of contributing data.

# Dissemination Policy

This study will be written up as a separate qualitative paper and as part of the RCT and submitted to peer reviewed journals as soon as possible. The previous INFORM study, undertaken by the same group, was published in the Lancet and other high impact journals. The work will also be presented at national and international conferences. The funders, sponsors and ethical review boards will be acknowledged in the papers. Individual clinicians must undertake not to submit any part of their individual data for publication without the prior consent of the TMG.

Both GHP and the University of Liverpool have active collaborations with the World Health Organization. These relationships will facilitate the consideration of the results of the trial in the development of clinical guidance and guidelines. The University of Liverpool is a WHO Collaborating Centre for Research and Research Synthesis in Reproductive Health, with Professor Weeks as the Director of this Centre. Professor Zarko Alfirevic, co-investigator is a member of the National Institute Health and Care excellence (NICE) induction of labour guideline committee, and co-ordinating editor of the Cochrane Collaboration Pregnancy and Childbirth Group. Professor Beverly Winikoff sits on the advisory committee of the International Federation of Gynaecology and Obstetrics (FIGO). FIGO has access to all of the national professional societies of obstetrics and gynaecologists. In addition, Dr Shuchita Mundle is a member of the Indian professional society, FOGSI. Professors Weeks, Winikoff and Alfirevic are on the FIGO expert group on misoprostol dosages.

# Authorship Eligibility Guidelines

Authorship will be attributed according to the amount of work undertaken on this project, currently it is planned that Dr Kate Lightly will be the first author. Co-authors will include other members of the q MOLI team including Dr Mundle and the MOLI RCT team. Professor Andrew Weeks is the Chief Investigator.

# References

[1] S. Mundle *et al.* “Foley catheterisation versus oral misoprostol for induction of labour in hypertensive women in India (INFORM): a multicentre, open-label, randomised controlled trial,” *Lancet*, vol. 390, no. 10095, pp. 669–680, Aug. 2017.

[2] WHO, UNICEF, UNFPA, World Bank Group and the U.N.P.D. “Trends in Maternal Mortality: 1990 to 2015”. Geneva, World Health Organization, 2015.

[3] H. Wang *et al.* “Global, regional, and national life expectancy, all-cause mortality, and cause-specific mortality for 249 causes of death, 1980–2015: a systematic analysis for the Global Burden of Disease Study 2015,” *Lancet*, Oct 8;388(10053):1459-1544.2016.

[4] J.P Vogel *et al.* “Global Perspectives on Elective Induction of Labor,” *Clin. Obstet. Gynecol.*, vol. 57, no. 2, pp. 331–342, Jun. 2014.

[5] World Health Organization. “Reproductive Health. Managing complications in pregnancy and childbirth: a guide for midwives and doctors". 2nd edition. World Health Organization; 2017.

[6] World Health Organization. “WHO recommendations for induction of labour". Geneva: World Health Organization; 2011.

[7] Polit DF, “Nursing research: generating and assessing evidence for nursing practice". 10th ed.Philadelphia (PA): Lippincott, Williams &amp; Wilkins; 2017.

[8] A. Moser *et al*. “Series: Practical guidance to qualitative research. Part 1: Introduction,” *Eur. J. Gen. Pract.*, vol. 23, no. 1, pp. 271–273, 2017.

[9] M.A Godhino *et al.* “Completeness of reporting in Indian qualitative public health research: a systematic review of 20 years of literature,” *J. Public Health (Bangkok)*, pp. 1–7, 2018.

[10] C. A. Corbett and L. C. Callister, “Giving Birth,” *MCN, Am. J. Matern. Nurs.*, vol. 37, no. 5, pp. 298–305, Sep. 2012.

[11] Z. Matthews *et al.* “Birth rights and rituals in rural south India: care seeking in the intrapartum period.” *J. Biosoc. Sci.*, vol. 37, no. 4, pp. 385–411, Jul. 2005.

[12] S. Bhattacharyya *et al.*  “Do women’s perspectives of quality of care during childbirth match with those of providers? A qualitative study in Uttar Pradesh, India,” *Glob. Health Action*, vol. 11, no. 1, p. 1527971, Jan. 2018.

[13] S. G. Bruce *et al.* “Preferences for infant delivery site among pregnant women and new mothers in Northern Karnataka, India,” *BMC Pregnancy Childbirth*, vol. 15, no. 1, p. 49, Dec. 2015.

[14] S. Rivenes Lafontan *et al.* “I Was Relieved to Know That My Baby Was Safe’: Women’s Attitudes and Perceptions on Using a New Electronic Fetal Heart Rate Monitor during Labor in Tanzania,” *Int. J. Environ. Res. Public Health*, vol. 15, no. 2, p. 302, Feb. 2018.

[15] S. Lou *et al.*, “Women’s experiences of postterm induction of labor: A systematic review of qualitative studies.” *Birth*, Dec. 2018.

[16] R. Coates *et al.* “Women’s experiences of induction of labour: Qualitative systematic review and thematic synthesis,” *Midwifery*. 2019.

[17] O.O. Enabor *et al.* “Cervical ripening and induction of labour-awareness, knowledge and perception of antenatal attendees in Ibadan, Nigeria.” *J. Obstet. Gynaecol.*, vol. 32, no. 7, pp. 652–6, Oct. 2012.

[18] M. Ezeanochie *et al.* “Women’s concerns and satisfaction with induced labour at term in a Nigerian population.,” *Niger. Postgrad. Med. J.*, vol. 20, no. 1, pp. 1–4, Mar. 2013.

[19] J. P. Vogel *et al* “Pharmacological and mechanical interventions for labour induction in outpatient settings,” *Cochrane Database Syst. Rev*, vol. 9, p. CD007701, Sep. 2017.

[20] S. Miller *et al.*, “Beyond too little, too late and too much, too soon: a pathway towards evidence-based, respectful maternity care worldwide,” *Lancet*, vol. 388, no. 10056, pp. 2176–2192, Oct. 2016.

[21] B.G Glaser and Strauss. “The discovery of grounded theory,” *Int. J. Qual. Methods*, 1967.

[22] N.K. Gale *et al*. “Using the framework method for the analysis of qualitative data in multi-disciplinary health research,” *BMC Med. Res. Methodol,* vol. 13, no. 1, p. 1, 2013.

[23] D.C.&N.C.B. Phillips, “Postpositivism and Educational Research,” Lanham & Boulder: Rowman & Littlefield Publishers, 2000.

[24] A Bryman. Social Research Methods. Oxford University Press, 2013.

[25] S.A. Hays. Qualitative Inquiry in Clinical and Educational Settings*.* New York: The Guilford Press, 2012.

[26] N.K Gale *et al.* “Using the framework method for the analysis of qualitative data in multi-disciplinary health research,” *BMC Med. Res. Methodol,* vol. 13, no. 1, p. 117, Dec. 2013.

# Appendices

## Appendix 1- Required Documentation

Qualitative protocol, including interview guides

Patient information sheets

Patient consent form

RCT protocol

CVs all researchers

Contracts U of L, Gynuity, LSTM, GMC, Daga

Ethics approval – GMC, Daga, U of L

## Appendix 2 – Schedule of Procedures for q MOLI
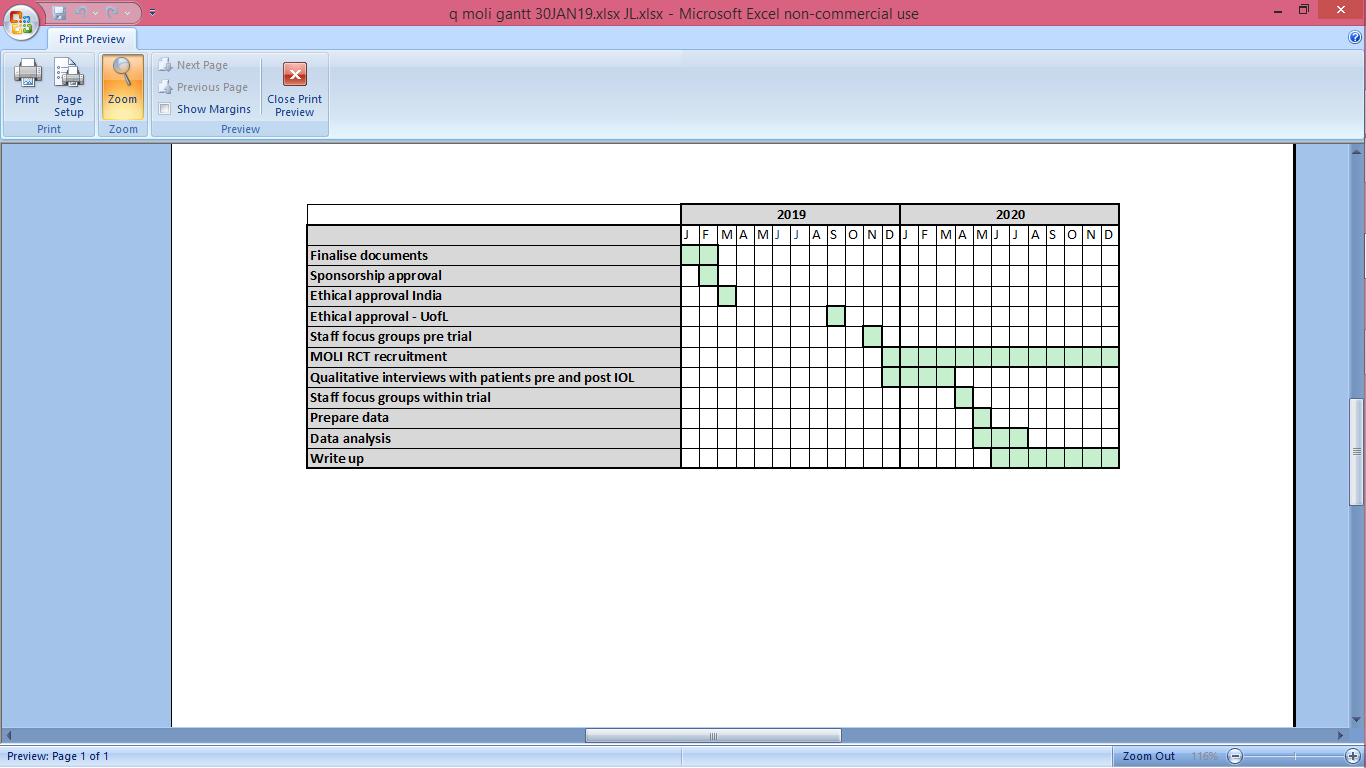


## Appendix 3 – Schedule of Procedures for RCT

| **Procedures** | **Screening and study RCT entry** | **Recruitment to randomised trial** | **24 hours after birth** | **Data taken from case notes after discharged End of trial** |
| --- | --- | --- | --- | --- |
| Assessment of eligibility criteria | x | x |  |  |
| Signed consent form | x |  |  |  |
| Qualitative interview (n=12 pre IOL, n=24 post IOL) | x |  | x |  |
| Randomisation |  | x |  |  |
| Administration of study intervention |  | x |  |  |
| Birth |  | x |  |  |
| Maternal complications |  | x | x | x |
| Fetal / neonatal complications |  | x | x | x |
| Serious maternal complications |  | x | x | x |
| Cost effectiveness |  |  |  | x |
| Efficacy of induction process |  |  | x |  |
| Assessment of adverse events |  |  | x | x |
| Mother Generated Birth Satisfaction Index | x |  | x |  |
| Participant Satisfaction Questionnaire |  |  | x |  |

##

## Appendix 4 – Amendment History

| **Amendment No.** | **Protocol version no.** | **Date issued** | **Author(s) of changes** | **Details of changes made** |
| --- | --- | --- | --- | --- |
| .01 | 1.01 | 18.5.20 | K Lightly | \| Sentence added to study timeframes.  *A few months after study commencement, MOLI and q MOLI study recruitment was halted due to the COVID pandemic. Although the initial focus groups had taken place, the follow-up data collection (planned for 4-6 months after the start of recruitment) had not yet taken place. As a result of ongoing uncertainty, the study period was extended to December 2021.* \| \| --- \| |

## Appendix 5 – MOLI Interview Guide 1: Women, pre IOL

The interviewer will ensure that the participant has consented and is willing to continue. The patient and researchers will move to a private room on the ward. The interviewer will thank the participant for agreeing to be interviewed and will attempt to make them feel as relaxed as possible.

To achieve the study objectives following the semi-structured schedule will be followed. The discussion will be participant-led with prompting questions from the interviewers.

Potential prompts are written in brackets, in italics, which can be used if the patient is not forthcoming with their responses. These do not have to be used, but could help the participant share more information if they are shy.

**Setting of ground rules**

Explain:

- The study and why it is being done
- Tape recording and transcription
- Study numbers/confidentiality
- Use of interpreter, if necessary
- That the participant can stop at any time
- That the participant can refuse to answer questions
- Explain opportunity to ask questions at any time
- Encourage participant be talkative and share their views as much as possible

Check consent form is signed, and the patient is happy to proceed

Check both tapes and start recording

**Interview guide**

Open by stating that we are trying to find out about how women feel about induction of labour, any previous experiences of it and what they know about it. So, we understand that your doctor has recommended induction of labour for you and you are already recruited to the MOLI trial.

- Can you tell me what induction of labour is and what you understand about it? *(Having medications to start labour, what does it mean? Have you heard of it before? What did your doctor say about it to you?)*
- Please can you describe to me the process of labour induction?

*(What will they do to help start your labour? Will they give you drugs? Which ones? Any other methods?)*

- Can you explain why are you having an induction of labour?

*(Having medications to start labour, do you have a medical condition? What is it? What did the doctor say?)*

- How do you feel about it?

*(In your own words tell me what you think about being induced/having medicine to start labour)*

- Can you explain what things are important to you about this process?

*Different things are important to different people and we would like to know what you think, it could be anything from timings, to safety, to place of birth, to the care you receive, to your family’s thoughts, just what you think)*

- Of these things (mentioned in last question), what is the most important thing?

*(The one thing that is the most important thing to you, it could be anything. If you had to choose one aspect, which would it be)*

- Is there anything that you feel particularly positive/happy about this process?

*(Things you were glad about or liked)*

- Is there anything that makes you feel particularly concerned/worried about this process?

*(Things that you didn’t like or made you sad/unhappy/sad about or things that could be better)*

- What do your family members/husband/think about this process?

*(Is anyone in the hospital with you now? What do they think/feel about you being induced?)*

- Have you ever been induced before/any of your relative/friends been induced before?

*(Had the drugs to start labour off, your friends? mother? sisters?)*

- What was your experience/their experiences like? Can you describe them?

*(How did they find it? We’re they happy or sad about it? What do you remember them telling you about it)*

- What do you think being induced will be like for you?

*What do you expect will happen? How do you think you will feel?*

- Can you tell me what you know about how your baby will be monitored (looked after) during labour?

*Will they listen to the babies heart beat during labour? How will they do this? Why do they do this? Is it important?*

- Can you tell me about the different types of fetal heart monitoring?

*(e.g. intermittent auscultation with Pinnard/Doppler or CTG intermittent/continuous)*

- What is important to you about monitoring the baby’s heart beat during labour?

*(How often, how long, straps around your tummy, who does it, why they are doing it, if its normal etc)*

- Do you have any preferences about how your baby is monitored? Why?

*Do any of these methods seem better than others? Or worse? Why? Do any methods appeal to you? Do any methods concern you? Why?*

- In some places around the world staff and women worry that listening to the babies heart beat all of the time in labour, using electronic monitoring, could mean extra false alarms and this means increases in interventions such as caesarean sections, unnecessary tests, or forceps deliveries, which are not needed. How do you feel about this?

*Does that worry you? Is it important to you?*

- If you could choose, how would you like to give birth? *(CS, forceps, normal)* And why?
- As part of the study paperwork you completed the Mother Generated Birth Satisfaction Index, (show the CRF) how did you feel about this process?

*Was it clear? Was it confusing? Was it too long? Or too short? Was it hard to think of the answers?*

- Do you feel that what was written on the form reflects your current thoughts about birth?

*Do the answers on the form reflect your honest thoughts and priorities?*

- Is there anything you want to add or tell me about that hasn’t been discussed?

After completion of the interview/discussion, ask the participant if they have any questions, thank them for their time and stop the recording.

## Appendix 6 – MOLI Interview Guide 2: Women, postnatal

The interviewer will ensure that the participant has consented and is willing to continue. The mother and child must also be settled and feeling well enough for the interview. The patient and researchers will move to a private room on the ward. The interviewer will thank the participant for agreeing to be interviewed, congratulate her on the birth of her child and will attempt to make them feel as relaxed as possible.

To achieve the study objectives following the semi-structured schedule will be followed. The discussion will be participant-led with prompting questions from the interviewers.

Potential prompts are written in brackets, in italics, which can be used if the patient is not forthcoming with their responses. These do not have to be used, but could help the participant share more information if they are shy.

**Setting of ground rules**

Explain:

- The study and why it is being done
- Tape recording and transcription
- Study numbers/confidentiality
- Use of interpreter
- That the participant can stop at any time
- That the participant can refuse to answer questions
- Explain opportunity to ask questions at any time
- Encourage participant be talkative and share their views as much as possible

Check consent form is signed, and the patient is happy to proceed

Check both tapes and start recording

**Interview guide**

Open by stating that we want to explore women’s understanding of and feelings towards induction of labour.

We understand that you were a participant in the MOLI trial and your labour was induced.

- Please can you tell me about the induction process and labour and how it was for you? What happened?

*(Can you remember when you were first given medicines to start labour, how did you feel, what happened next, then what happened…)*

- Do you know which induction method you had?

*(Breaking waters, tablet/tablet or tablet/hormone drip)*

- How do you feel about the induction in general?

*(In your own words tell me what you think about being induced/having medicine to start labour, did you like/dislike the idea)*

- Was the process acceptable for you overall?

*(All in all, did the induction and labour go ok? When you look back on it, how did it go)*

- Is there anything that you feel particularly positive/happy about this process?

*(Things you were glad about or liked)*

- Is there anything that makes you feel particularly concerned/worried about this process?

*(Things that you didn’t like or made you sad/unhappy/sad about or things that could be better)*

- How did going through the induction process compare to what you thought it would be like before the induction?

*(Was having the medicines like how you imagined it to be? Was it better or worse? Or had you not really thought about it? Which aspects were different)*

- Before you had your baby, what were the most important things to you about the process?

*(Tell me all of the things that were important to you before you had your baby, of these which was the most important or the thing you cared about the most?)*

- What is the most important thing to you now, about this induction process, now that you have gone through it and you have had your baby?

*(Tell me all of the things that are important to you now, of these which is the most important or the thing you care about the most?)*

- Is what is important to you now after the birthing process, the same as what was important to you before you had your baby? How has your view changed?

*(Tell me all of the things that are important to you, are these the same now that you have had your baby?)*

- Was there anything especially good about the induction method that you had?

*(Things you were glad about or liked about the tablets/drip)*

- Was there anything bad/that you did not like about the induction method that you had?

(*Things that you didn’t like or made you sad/unhappy/sad about or things that could be better about the tablets/drip)*

- Would you use this method (tablets/drip) again if you had the choice? Why?

*(If you had to be induced again, would you choose to have the tablets or the drip when you have your next baby, what did you like/not like about it?)*

- Would you recommend this method to a friend/family member? Why?

*(If you were advising your friend or family member, which medicine would to suggest? tablets/drip?, what did you like/not like about it?)*

- If you could choose to have the drip or the tablets throughout labour, which would you prefer? Why?

*(Tablets/drip, what did you like/not like about it?)*

- Which way was your baby born? (caesarean/forceps/normal)
- How do you feel about this?

*(Happy, sad, indifferent)*

- Which way do you think is the best way for a baby to be delivered? (caesarean/forceps/normal) Why?

*(If you could choose which way to deliver a baby, which way is the best?)*

- Can you describe to me how your baby monitored during the labour? *(Describe hand held device IA and CTG machines)*
- How often was it done?

*(Regularly, every hour, never, all of the time, 2x in labour 20x in labour)*

- How did you feel about this fetal monitoring?

*(Happy, sad, indifferent, did you notice it?)*

- Do you know of any other ways of monitoring the baby in labour?

*(Describe hand held device and CTG machines)*

- Was there anything good about it?

*(Monitoring the babies heart beat)*

- Was there anything that could have been better about it or you didn’t like?

(*Things that you didn’t like or made you sad/unhappy/sad about or things that could be better about the tablets/drip)*

- What fetal monitoring would you ideally like if you could choose?

*(Describe hand held device and CTG machines)*

- As part of the study paperwork you completed the Mother Generated Birth Satisfaction Index, (show the CRF) how did you feel about this process?

*Was it clear? Was it confusing? Was it too long? Or too short? Was it hard to think of the answers?*

- Do you feel that what was written on the form reflects your current thoughts about birth?

*Do the answers on the form reflect your honest thoughts and priorities?*

- Is there anything you want to add or tell me about that hasn’t been discussed?

After completion of the interview/discussion, ask the participant if they have any questions, thank them for their time and stop the recording.

## Appendix 7 – MOLI Interview Guide 3: MOLI Interview Guide: Staff focus groups – before start of trial

The researcher will ensure that the participants are happy to join the focus group and understand that this is voluntary. The interviewer will thank the participants for agreeing to join the focus group and attempt to make them feel as relaxed as possible. It is important to emphasise that any information disclosed is confidential, unless there is any potential patient harm from anything disclosed and that openness and sharing of views is essential, in order to maximise the information gathering potential of the focus group.

**Setting of ground rules**

Explain:

- The study and why it is being done
- Tape recording and transcription
- Study numbers/confidentiality
- That the participants can stop/leave at any time
- That the participants can refuse to answer questions
- Explain opportunity to ask questions at any time

Check consent forms are signed

Check both tapes and start recording

**Interview guide**

This aim of this focus group is to gather information from the staff involved in screening, recruiting, randomising and consenting for the MOLI trial in order to gather as much information as possible surrounding the trial and the different regimes. We would like to hear your honest views, so that we can understand the results of the trial better.

Research generally (ice breaker)

Tell me about your experiences of taking part in the trial MOLI Trial so far

Do you feel it will be acceptable to patients?

Do you have concerns/worries about the trial/these protocols?

Can you forsee any potential barriers to recruitment?

Can anyone suggest any potential solutions for any problems?

Can you highlight any areas for improvement?

Tell me about your previous experiences of induction of labour

Any positive?

Any negatives?

How do patients find induction of labour?

What is the hardest part of induction for women?

In this trial there are two treatments, one is a misoprostol/oxytocin regime

Do you have any experience of using this regime?

What were your previous experiences of using the miso/oxytocin regime?

Any positive?

Any negatives?

How do patients find it?

The other treatment in this trial is misoprostol/misoprostol regime, using misoprostol from the start of induction to birth

Do you have any previous experiences of using the miso/miso regime?

Do you have concerns/worries about using it?

How do you think it will work logistically?

As part of the MOLI trial, we plan to have more CTG machines available on labour ward and to do some training about fetal monitoring in labour

What do you currently use for fetal monitoring in your hospital?

How well does this work?

Are there any good things about it?

Any bad things about it?

How do you feel about having CTGs on labour ward?

Do you feel it will change patient care?

How?

Are there any good things about it?

Any bad things about it?

Internationally there is concern that introducing CTG could increase the intervention rate e.g. increase the caesarean rate. How do you feel about this?

Is there anything you want to add or tell me about that hasn’t been discussed?

After completion of the focus group, ask the participants if they have any questions, thank them for their time and switch off the recording.

## Appendix 8 - MOLI Interview Guide 4: Staff focus groups during trial

The researcher will ensure that the participants are happy to join the focus group and understand that this is voluntary. The interviewer will thank the participants for agreeing to join the focus group and attempt to make them feel as relaxed as possible. It is important to emphasise that any information disclosed is confidential, unless there is any potential patient harm from anything disclosed and that openness and sharing of views is essential, in order to maximise the information gathering potential of the focus group.

**Setting of ground rules**

Explain:

- The study and why it is being done
- Tape recording and transcription
- Study numbers/confidentiality
- That the participants can stop/leave at any time
- That the participants can refuse to answer questions
- Explain opportunity to ask questions at any time

Check consent forms are signed

Check both tapes and start recording

**Interview guide**

This aim of this focus group is to gather information from the staff involved in screening, recruiting, randomising and consenting for the MOLI trail in order to gather as much information as possible surrounding the trial and the different regimes. We would like to hear your honest views, so that we can understand the results of the trial better.

MOLI Trial

How do you feel the trial has gone so far?

Has anything gone particularly well/you liked?

Do you have concerns/worries about the trial?

Is there anything that could have been done better?

Tell me about your previous experiences of induction of labour

Any positive?

Any negatives?

How do patients find induction of labour?

What is the hardest part of induction for women?

In this trial there are two treatments, one is a misoprostol/oxytocin regime

What are your experiences of using the miso/oxytocin regime?

Any positive aspects/benefits for patients or staff?

Any negative aspects for patients or staff?

Do you think it works well?

How do patients find it?

Suggestions for improvements?

The other treatment in this trial is misoprostol/misoprostol regime, using misoprostol from the start of induction to birth

What were your experiences of using the miso/miso regime?

Any positive aspects/ benefits for patients or staff?

Any negative aspects for patients or staff??

How do patients find it?

Barriers for implementation if found to be better?

Suggestions for improvements?

If you/your relative/friend had to be induced, which regime would you prefer and why?

As part of the MOLI trial, we plan to have more CTG machines available on labour ward and to do some training about fetal monitoring in labour

Tell me about the different types of fetal monitoring available on labour ward currently

Which fetal monitoring do you prefer and why?

How well does this work?

Have you noticed that there are now more CTG machine on labour ward?

How do you feel about having more CTG machines on labour ward?

Do you feel it has changed patient care at all?

How?

Has it had any good changes/impacts?

Has it had any bad changes/impacts?

Internationally there is concern that introducing CTG could increase the intervention rate e.g. increase the caesarean rate. How do you feel about this?

How do you feel about the Mother Generated Birth Satisfaction Index used?

Is there anything you want to add or tell us about that hasn’t been discussed?

After completion of the focus group, ask the participants if they have any questions, thank them for their time and switch off the recording.

## Appendix 9 – Patient consent form

## Appendix 10 – Patient information leaflet

## Appendix 11 – MOLI Randomised Controlled Trial Protocol
