# Supplementary material for: Evaluating women’s experiences and satisfaction with labour induction in India: a comparison of the participant generated experience and satisfaction (PaGES) index with standard methods
Source: BMC Pregnancy Childbirth. 2025 May 28;25:619. doi: 10.1186/s12884-025-07731-9 (PMC12117871; doi:10.1186/s12884-025-07731-9)
Supplement: Supplementary file 2 — Supplementary Material 2 [file 12884_2025_7731_MOESM2_ESM.docx]

**Appendix 1.** Summary of existing tools to measure birth experience and satisfaction. These tools have been widely utilised and validated.

| **Satisfaction and experience tool** | **Study purpose** | **Overview** | **Format of questionnaire/tool** |
| --- | --- | --- | --- |
| **Six Simple Questions** (15) | To assess the differences in women's satisfaction with maternity care from doctors and midwives | Canada  194 women randomly assigned to receive either midwife care or doctor care had satisfaction measured at 36 weeks gestation, 48hrs postpartum, 2 weeks postpartum and 6 weeks postpartum. | 6 item questionnaire scored using 7-point Likert scale. |
| **Perceptions of Care Adjective Checklist** (14) | To understand women’s experience of maternity care | England  4800 women randomly selected for sampling at three months postpartum. 2960 responses used in analysis. | 15 terms (7 positive and 8 negative) were listed, and women could select any number of these. |
| **The Childbirth Experience Questionnaire** (44) | To assess various aspects of first-time mother’s perception of childbirth experience | Sweden  1177 primiparous women completed questionnaire one month postpartum within a study on labour progression and oxytocin. | 22 item questionnaire scored using 4-point Likert scale. |
| **The Responsiveness in Perinatal and Obstetric Health Care Questionnaire** (45) | To evaluate maternal experiences of perinatal care services | The Netherlands  274 women invited for study participation two weeks postpartum. 171 interviews used in analysis. | Survey administered via face-to-face 20-40 minute interviews.  Questioned according to eight-domain WHO concept; dignity, autonomy, confidentiality, communication, attention, social consideration, quality of amenities and choice/continuity of health care provider. |
| **Pregnancy and maternity care patients experiences questionnaire** (46) | To measure women’s experiences of pregnancy, childbirth and postpartum healthcare | Norway  8670 women invited to complete the interview 17 weeks postpartum. 4904 questionnaires returned. | 145 item questionnaire answered using a 5-point Likert scale. |

| **The Childbirth Perception Scale** (47) | To assesses perception of delivery and early postpartum phase | The Netherlands  1347 pregnant women invited to participate. 978 women completed the questionnaire after delivery. | 12 item questionnaire with two domains (perception of delivery and perception of first week postpartum). |
| --- | --- | --- | --- |
| **Wijma Delivery Expectancy/experience Questionnaire** (12) | To measure fear of childbirth during pregnancy and following childbirth | Sweden  196 pregnant women competed the first questionnaire at 32 weeks gestation. Of these 166 completed the second form within two hours of delivery and 175 completed the third form at five weeks postpartum. | 33 item questionnaire answered using a 6-point Likert scale. |

**Appendix 2.** Completed antenatal PaGES case report form

**
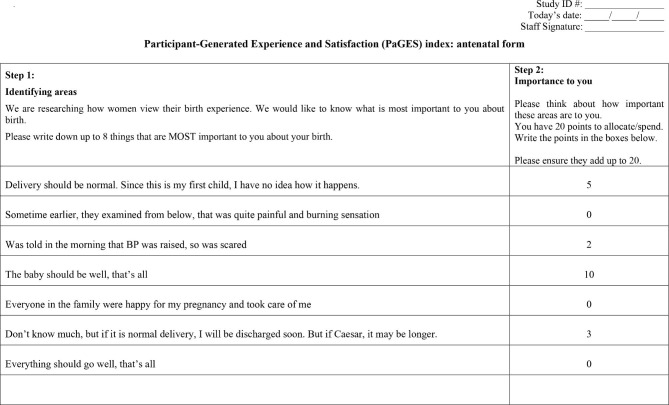
**

**Appendix 3a. “**Women’s experiences of IOL and childbirth” (subthemes and illustrative qMOLI quotes).

| **Subthemes** | **Illustrative quotes** |
| --- | --- |
| *Blood pressure* | “Only was worrying about BP.” **(Interview 3)** |
| *Childbirth* | “Doctor said time has come. As soon as I got there, I had to push hard. Doctor was asking me to push, I tried, I had a lot of strength (smiled), I pushed hard, and it delivered normally.” **(Interview 31)** |
| *Environment* | “I was seeing the ladies because of that I was more scared.” **(Interview 11)**  “I had never seen Medical in my whole life […] One thought was, how will I do here? Because the environment is not good. […] Obviously, it will get dirty as so many people are there.” **(Interview 51)** |
| *Family* | “[…} family members were too upset.” **(Interview 18)**  “My family members are happy. Specially my husband is very happy.” **(Interview 46)**  “My family members were also afraid that she should deliver a baby nicely.” **(Interview 48)** |
| *IOL process* | “I felt nothing after taking the two pills. I was in a bit of pain, after taking the third pill. After that, they applied the saline. Then it was paining even more painful. Couldn't bear. When saline was finished then it was paining even more, and I thought I will survive or not.” **(Interview 31)**  “They had given the first pill, I took that. It didn't bring the pain. Given a second pill, there was a backache. After the second pill, I had more pain.” **(Interview 37)** |
| *Knowledge* | RA- “Did your mother told you about induction of labour, etc.?” P- “No. She doesn’t know anything like this.” RA- “You, have you heard from anywhere this?” P- “No. I don't. Didn't know.” **(Interview 10)**  “No. I don't have knowledge about it… but I heard from my friend, she also delivered normally. She told me, "I went to the hospital, and I delivered normally within half an hour ". I heard that. Then, I was confirmed that when she would deliver normally then why not me?” **(Interview 45)** |
| *Pain / ‘traas’* | “When there was pain at that time means I was feeling that now I can't survive. (Feared expression on the face with a smile).” **(Interview 23)**  “What a pain… the pain was so much… it should come in 5 minutes, I was feeling it's coming every 2-3 minutes... such a pain was there, a lot of pain, lot of pain.” **(Interview 46)** |
| *Staff* | “And then one Sapna madam was there. She helped me a lot. She checked my BP frequently. Made me listen to my baby's heart beats. Gave me the pill. Asked me to walk frequently. Told lots of times. Means, because of her I walked.” **(Interview 46)** |
| *Thoughts and feelings* | “Nervousness is there na […] How will it happen, what will happen? Whether it would be normal or not?” **(Interview 18)**  “And it was my first time na. So, I was feeling that someone didn't even conceive. At least we have, that's okay. Whatever will happen, will happen by the grace of God. These were the thoughts in mind before.” **(Interview 23)**  “I am happy. Happy means my delivery occurred earlier.” **(Interview 49)** |

**Appendix 3b.** “Important areas identified by women” (subthemes and illustrative qMOLI quotes).

| **Subthemes** | Illustrative quotes |
| --- | --- |
| *Baby’s future* | “Yes... like I became a mother... how will be my baby? What will he do in future? What will he not do? These things I told you.” **(Interview 37)**  “Now my baby should remain well.” **(Interview 48)** |
| *Baby’s gender* | “There was no tension, whether it's a baby girl or male.” **(Interview 3)**  “My family members are happy. Specially my husband is very happy. He wanted a girl.” **(Interview 46)**  “I was very happy that it's a baby boy. My father-in-law has also wanted a baby boy since first. I am a lovable daughter in law (laughed).” **(Interview 52)** |
| *Early birth and discharge* | “Now, waiting to go home. When it will discharge (smiled).” **(Interview 10)**  “Should deliver early, that's only.” **(Interview 48)**  “I asked madam, how long will my delivery take or will it take more time. Earlier also it took much time.” **(Interview 49)** |
| *Mode of birth* | “Caesar don't have trouble early but afterwards there is a lot of trouble. For that, normal delivery is good.” **(Interview 8)**  “Normal is okay only [...] Normal only means nothing, there is not so much trouble.” **(Interview 23)**  “No, I am very scared of Caesar. Because I heard from lots of people that in Caesar there is not so much trouble first but when it's there, it will never go.” **(Interview 45)**  “I want to do only Caesar. I don't want to do normal. I was saying no means no.” **(Interview 51)** |
| *No pain or ‘traas’* | “I was feeling that there should not be much pain. Pain should come but not much.” **(Interview 10)**  “I thought there should not be more trouble. I ate a tablet so there was not so much trouble. Trouble was there at the time of the baby's arrival only.” **(Interview 48)** |
| *Own health* | “My BP should not raise. I feel this. I have BP na… BP should not raise. Feel that my health is good.” **(Interview 10)**  “This hospital means, I asked him (husband) to do it in private. So that my operation (family planning) can also be done na.” **(Interview 16)** |
| *Safety and health of baby* | “Only thing I was thinking that my baby should be safe. Importantly, I could have done anything for that.” **(Interview 16)**  “P- No, only the baby is important. Now, she is good. Not drinking milk now. Unable to hold.” **(Interview 46)** |

**Appendix 4.** Table 4 extended showing Likert, PaGES and qMOLI responses for 20 randomised participants who completed all three tools. Likert scores and overall PaGES scores are colour coded - positive (green), neutral (yellow) and negative (red).

| **ID (qMOLI ID)** | **Group** | **Likert questions** | | | | **PaGES Index** | | | | **qMOLI** | **Interpretation** |
| --- | --- | --- | --- | --- | --- | --- | --- | --- | --- | --- | --- |
|  |  | **Augmentation** | **Delivery time** | **Pain** | **Anxiety** | **PP statements** | **Beans** | **Satisfaction** | **Overall Score** |  |  |
| 10266 (3) | Oxy | 4 | 5 | 4 | 4 | Happy – general | 10 | 8 | 154 | “There was no tension, whether it's a baby girl or male.”  “Caesar done? Hurt for a while. Now, doesn't feel anything.”  “Only was worrying about BP.” | Very unacceptable Likert delivery time score but not mentioned in qMOLI interview. |
|  |  |  |  |  |  | Family – happy/good | 2 | 7 |  |  |  |
|  |  |  |  |  |  | Gender – Important | 6 | 8 |  |  |  |
|  |  |  |  |  |  | MOB – dissatisfaction with CS | 0 | 3 |  |  |  |
|  |  |  |  |  |  | Postpartum – general | 0 | 4 |  |  |  |
|  |  |  |  |  |  | Postpartum – now I am good | 0 | 4 |  |  |  |
|  |  |  |  |  |  | Pain general | 2 | 6 |  |  |  |
| 10320 (4) | Oxy | - | 4 | 4 | 4 | Gender – Important | 20 | 10 | 200 | “Wanted to do delivery quickly… Because the trouble was so much.”  “Important? Baby only.” | Low Likert scores but gave 10/10 satisfaction with all PP PaGES statements. |
|  |  |  |  |  |  | Physical symptoms | 0 | 10 |  |  |  |
|  |  |  |  |  |  | Long-term future – baby | 0 | 10 |  |  |  |
|  |  |  |  |  |  | MOB – satisfaction with NVD | 0 | 10 |  |  |  |
|  |  |  |  |  |  | MOB – satisfaction with NVD | 0 | 10 |  |  |  |
|  |  |  |  |  |  | Gender – family important | 0 | 10 |  |  |  |
| 20072 (8) | Miso | 2 | 3 | 4 | 4 | Gender – important | - | 10 |  | “Whatever it may be, girl or boy it doesn't matter to us.”  “Caesar don't have trouble early but afterwards there is a lot of trouble. For that, normal delivery is good.”  “Important is baby only. It changes the whole life.”  “After seeing all women’s delivery... they were screaming, doing that. Therefore, I scared most... because of that, my BP raised.”  “How it will happen, what will happen? The fear of this was most.” | High Likert anxiety score could be due to environment as described in qMOLI interview.  Gender important in PaGES but not qMOLI. |
|  |  |  |  |  |  | Delivery – environment | - | 0 |  |  |  |
|  |  |  |  |  |  | Postpartum – now I am good | - | 9 |  |  |  |
|  |  |  |  |  |  | Baby – healthy | - | 10 |  |  |  |
|  |  |  |  |  |  | MOD – satisfaction with NVD | - | 10 |  |  |  |
| 20080 (9) | Oxy | 2 | 2 | 3 | 4 | Postpartum – now I am good | - | 10 |  | “I never done discrimination between boy and girl… if there had been another girl, I would be happy.”  “The baby should be good, normal. Now, my baby is in glass. He should be healthy. Should be good.”  “My BP was raised.” | Very positive Likert and PaGES satisfaction scores except for anxiety. |
|  |  |  |  |  |  | Misc – general | - | 10 |  |  |  |
|  |  |  |  |  |  | Family – happy/good | - | 10 |  |  |  |
|  |  |  |  |  |  | Postpartum – now I am good | - | 10 |  |  |  |
|  |  |  |  |  |  | Postpartum – family planning | - | 10 |  |  |  |
|  |  |  |  |  |  | Happy – general | - | 10 |  |  |  |
| 10591 (10) | Oxy | 2 | 3 | 3 | 2 | Postpartum – now I am good | 2 | 8 | 114 | “Now, waiting to go home. When it will discharge (smiled).”  “There is lots of trouble if the caesar is done. Felt good as delivered soon. But I was expecting that my delivery should have been normal.”  “There should not be much pain. Pain should come but not much.”  “My BP should not raise… Both are important. Health and baby also.” | Happy with misoprostol augmentation method and delivery time.  Disappointment with CS in PaGES and qMOLI.  Discharge mentioned in qMOLI and PaGES (key priority) |
|  |  |  |  |  |  | Postpartum – pain | 0 | 4 |  |  |  |
|  |  |  |  |  |  | Gender – important | 3 | 6 |  |  |  |
|  |  |  |  |  |  | Gender – not important | 0 | 9 |  |  |  |
|  |  |  |  |  |  | MOB – dissatisfaction with CS | 10 | 3 |  |  |  |
|  |  |  |  |  |  | Postpartum – discharge | 0 | 8 |  |  |  |
|  |  |  |  |  |  | Postpartum – discharge | 5 | 10 |  |  |  |
| 10611 (11) | Miso | 2 | 3 | 3 | 2 | Gender – Important | 10 | 10 | 150 | “I am suffering because of caesar. But I am happy I had a baby girl.”  “Because of my BP I had to do ceasar.”  “I was even more nervous when I saw here.  There, I have seen all in one room. I was absolutely nervous. So, my BP even raised more.”  “Tablet was good. You have less pain because of that.” | Happy with misoprostol augmentation method. |
|  |  |  |  |  |  | MOB – dissatisfaction with CS | 5 | 5 |  |  |  |
|  |  |  |  |  |  | Postpartum – now I am good | 0 | 0 |  |  |  |
|  |  |  |  |  |  | Delivery – timeframe | 5 | 5 |  |  |  |
| 10665 (16) | Oxy | 1 | 2 | 2 | 3 | Everything was good – delivery | 5 | 8 | 175 | “I sincerely wanted to have a daughter.”  “The important things means that sir's (care)…and I joined in this (in study), therefore I benefited a lot. I think otherwise I would not have been delivered yet.”  “I asked him (husband) to do it in private. So that my operation (family planning) can also be done na.”  “Only thing I was thinking that my baby should be safe. Importantly, I could have done anything for that.” | Gender importance comes across in PaGES and qMOLI. |
|  |  |  |  |  |  | Everything was good – staff | 5 | 9 |  |  |  |
|  |  |  |  |  |  | Delivery – timeframe | 0 | 5 |  |  |  |
|  |  |  |  |  |  | Delivery - baby and had NVD | 10 | 9 |  |  |  |
|  |  |  |  |  |  | Gender – important | 0 | 6 |  |  |  |
|  |  |  |  |  |  | Postpartum – discharge | 0 | 5 |  |  |  |
| 20102 (18) | Miso | 2 | 4 | 4 | 4 | MOD - dissatisfaction with CS | 5 | 5 | 118 | “Baby should be good; mother should be healthy. That's it. After having a baby, the baby should remain good. Will think of this only na.”  “Family members were too upset. Like, why it's not happening yet.”  “Nervousness is there na… How will it happen, what will happen? Whether it would be normal or not?”  “That's only that in normal delivery all remains normal. And in Caesar there is a problem.” | High Likert anxiety score also discussed in qMOLI interview.  PaGES says family happy/good but in qMOLI family described as upset and frustrated.  Dissatisfaction with CS comes across in PaGES and qMOLI |
|  |  |  |  |  |  | Baby – healthy | 7 | 7 |  |  |  |
|  |  |  |  |  |  | Long-term future – general | 1 | 1 |  |  |  |
|  |  |  |  |  |  | Postpartum – now I am good | 1 | 8 |  |  |  |
|  |  |  |  |  |  | Family – happy/good | 3 | 10 |  |  |  |
|  |  |  |  |  |  | Misc – general | 1 | 1 |  |  |  |
|  |  |  |  |  |  | Family - happy/good | 2 | 2 |  |  |  |
| 20265 (23) | Miso | 2 | 3 | 4 | 3 | Baby – healthy | 10 | 10 | 190 | “Normal (delivery) is okay only.”  “It is important to start the labour pain.”  “They don't even tell me that it's like this happens at the time of caesar, it happens in normal. I was very much scared. How it will happen, what will happen? It is the first.”  “When there was pain na.. at that time means I was feeling that now I can't survive.” | Importance of labour pain.  Lack of knowledge. |
|  |  |  |  |  |  | Gender – important | 5 | 9 |  |  |  |
|  |  |  |  |  |  | Postpartum – pain | 0 | 2 |  |  |  |
|  |  |  |  |  |  | Everything was good – staff | 0 | 5 |  |  |  |
|  |  |  |  |  |  | Delivery – difficulty/trouble | 5 | 9 |  |  |  |
| 10847 (27) | Miso | 2 | 3 | 4 | 1 | Postpartum – general | 5 | 5 | 100 | “My baby delivered nicely. That only I feel good.”  “My baby should remain well. He is weak. He should get better.”  “BP was raised suddenly. Therefore, there was fear until the delivery.”  “I was saying ceasar earlier because there was lot of pain. But, now I feel that it's good that caesar is not done.” | High pain level reported in Likert and qMOLI. |
|  |  |  |  |  |  | Delivery – timeframe | 5 | 5 |  |  |  |
|  |  |  |  |  |  | Postpartum – now I am good | 0 | 5 |  |  |  |
|  |  |  |  |  |  | MOB – satisfaction with NVD | 5 | 5 |  |  |  |
|  |  |  |  |  |  | Baby – healthy | 0 | 5 |  |  |  |
|  |  |  |  |  |  | Baby – healthy | 2 | 5 |  |  |  |
|  |  |  |  |  |  | Blood pressure | 3 | 5 |  |  |  |
| 10894 (31) | Oxy | 2 | 2 | 3 | 2 | Postpartum – now I am good | 5 | 7 | 135 | “Now, it's a girl, so I'll educate her. I will educate her and she will be someone. This is my wish about my baby girl.”  “Therefore, I had that pill and that (saline). I also wanted to be delivered soon. And there should not be more pain also.”  “My baby should remain well, that's only important for me.”  “I was scared about labour pain. It was troubling a lot that time...a lot... can't tolerate.” | Positive Likert scores but lots of pain and anxiety discussed in qMOLI interview. |
|  |  |  |  |  |  | Misc – medication | 0 | 6 |  |  |  |
|  |  |  |  |  |  | Gender – important | 10 | 10 |  |  |  |
|  |  |  |  |  |  | Physical symptoms | 0 | 2 |  |  |  |
|  |  |  |  |  |  | Postpartum – discharge | 5 | 0 |  |  |  |
| 20583 (37) | Miso | 2 | 5 | 4 | 4 | Misc – motherhood | 5 | 10 | 125 | “How will be my baby? What will he do in future?”  “I wanted a boy. I delivered a girl.”  “My baby should be safe. Doctor should also tell everything is good.”  “They had given the first pill, I took that. It didn't bring the pain… After the second pill, I had more pain.” | Baby’s future discussed in PaGES and qMOLI. |
|  |  |  |  |  |  | Long-term future – baby | 5 | 5 |  |  |  |
|  |  |  |  |  |  | Postpartum - care for baby | 5 | 5 |  |  |  |
|  |  |  |  |  |  | Gender – not important | 5 | 5 |  |  |  |
| 21045 (45) | Oxy | 1 | 2 | 4 | 4 | Happy – general | 10 | 10 | 200 | “I am very scared of Caesar.”  “Family members and doctors explained to me a lot.”  “I don't have knowledge about it.”  “I couldn't sit, couldn't stand, there was a lot of pain.”  “I was very happy when I saw the baby for the first time. I was not feeling that there was pain. I forgot all the pain after seeing him.” | High pain level reported in Likert and qMOLI.  Happy to have a baby discussed in PaGES and qMOLI. |
|  |  |  |  |  |  | Happy – to have baby | 10 | 10 |  |  |  |
|  |  |  |  |  |  | Physical symptoms | 0 | 1 |  |  |  |
|  |  |  |  |  |  | Postpartum – breastfeeding | 0 | 2 |  |  |  |
| 21059 (46) | Miso | 2 | 3 | 4 | 4 | Postpartum – now I am good | 2 | 6 | 192 | “My husband is very happy. He wanted a girl.”  “Check the BP. Take good care. Gives medicine on time. All these processes are important.”  “Only the baby is important.”  “In the 9th month, I have been told that my BP is raised.”  “This pain was unbearable. I couldn't bear that.” | High pain level reported in Likert and qMOLI. |
|  |  |  |  |  |  | Postpartum – care for baby | 0 | 6 |  |  |  |
|  |  |  |  |  |  | Misc – motherhood | 10 | 10 |  |  |  |
|  |  |  |  |  |  | Gender – family important | 3 | 10 |  |  |  |
|  |  |  |  |  |  | Misc – medication | 5 | 10 |  |  |  |
| 21163 (47) | Oxy | 2 | 1 | 4 | 4 | Blood pressure | 0 | 5 | 200 | “My baby should remain well. Also, my daughter should remain well. My family members also should remain well and me also should remain well.”  “They were checking many times by inserting their hands. It was very painful. The pain could not be tolerated.” | Did not like vaginal examinations but augmentation given a positive Likert score. |
|  |  |  |  |  |  | Misc – medication | 10 | 10 |  |  |  |
|  |  |  |  |  |  | Happy – to have baby | 10 | 10 |  |  |  |
|  |  |  |  |  |  | Misc – general | 0 | 2 |  |  |  |
|  |  |  |  |  |  | Long-term future – baby | 0 | 8 |  |  |  |
| 11190 (48) | Miso | 2 | 3 | 4 | 4 | Misc – medication | 0 | 6 | 175 | “Means my husband wanted a baby girl. Means, his wish was first baby girl.”  “Should deliver early, that's only.”  “I wanted normal only.”  “I thought there should not be more trouble.  There was a lot of pain in the abdomen.” | Importance of family.  High pain reported across all three tools. |
|  |  |  |  |  |  | Pain – general | 2 | 3 |  |  |  |
|  |  |  |  |  |  | Gender – important | 5 | 9 |  |  |  |
|  |  |  |  |  |  | MOB – satisfaction with NVD | 3 | 8 |  |  |  |
|  |  |  |  |  |  | Gender – family not important | 0 | 7 |  |  |  |
|  |  |  |  |  |  | Happy – to have baby | 10 | 10 |  |  |  |
| 11189 (49) | Oxy | 2 | - | 4 | 5 | Blood pressure | 5 | 3 | 131 | “I asked madam, "how long will my delivery take".”  “I wanted my baby to come out safely… I thought that my baby should not be mentally retarded.”  “I had a problem with BP otherwise I didn't have that much trouble during labour pains.”  “I am happy. Happy means my delivery occurred earlier.” | Reports high pain on Likert form but says did not have much trouble with pain.  Delivery time discussed in qMOLI and PaGES, but no Likert score given. |
|  |  |  |  |  |  | Delivery – timeframe | 0 | 4 |  |  |  |
|  |  |  |  |  |  | Delivery – story of birth | 2 | 1 |  |  |  |
|  |  |  |  |  |  | Worried – baby | 0 | 2 |  |  |  |
|  |  |  |  |  |  | Gender – important | 3 | 8 |  |  |  |
|  |  |  |  |  |  | MOB – satisfaction with NVD | 10 | 9 |  |  |  |
| 11222 (50) | Oxy | 2 | 4 | 3 | 4 | MOB – had CS | 5 | 5 | 149 | “For induction of labour, I would like caesar only.”  “BP should not get raised. BP was too much raised. BP checking was going on. BP didn't get normal, therefore had to do the Caesar.”  “My delivery, my baby, was important for me.”  “It was very painful, madam. There was a lot of trouble. I regretted that I conceived.” | Unable to differentiate IOL from overall birth experience.  BP is a priority in PaGES and qMOLI. |
|  |  |  |  |  |  | Delivery – difficulty/trouble | 0 | 3 |  |  |  |
|  |  |  |  |  |  | Everything was good – all | 2 | 7 |  |  |  |
|  |  |  |  |  |  | Everything was good – me and baby | 3 | 10 |  |  |  |
|  |  |  |  |  |  | Postpartum – pain | 0 | 5 |  |  |  |
| 11254 (51) | Miso | 2 | 4 | 4 | 3 | Postpartum – pain | 0 | 7 | 180 | “Obviously, baby's health is important. Her future, her health should be good.”  “I want to do only Caesar. I don't want to do normal.”  “BP should not get raised.”  “The environment is not good. (hygiene, overcrowding).” |  |
|  |  |  |  |  |  | Delivery – difficulty/trouble | 0 | 6 |  |  |  |
|  |  |  |  |  |  | Baby – healthy | 10 | 9 |  |  |  |
|  |  |  |  |  |  | Gender – important | 10 | 9 |  |  |  |
|  |  |  |  |  |  | Everything was good – all | 0 | 9 |  |  |  |
|  |  |  |  |  |  | Postpartum – discharge | 0 | 9 |  |  |  |
| 21343 (52) | Oxy | 2 | 4 | 5 | 4 | MOB – had CS | 0 | 5 | 180 | “I was thinking, normal delivery will happen soon.”  “I wanted a normal delivery only. But it's fine if Caesar will happen.”  “Atmosphere is important. Now, it is not too cold, not too hot. I think this is good for the baby.”  “I am telling you; doctors didn't pay attention to anyone but me. I am very happy that they paid attention towards me.” | Baby is the priority. |
|  |  |  |  |  |  | Baby – most important | 10 | 10 |  |  |  |
|  |  |  |  |  |  | Misc – medication | 0 | 6 |  |  |  |
|  |  |  |  |  |  | Everything was good – me and baby | 5 | 10 |  |  |  |
|  |  |  |  |  |  | Worried – baby | 0 | 0 |  |  |  |
|  |  |  |  |  |  | Happy – to have baby | 0 | 10 |  |  |  |
